# Multiple-Mobile-Sink-Assisted Data Collection in Wireless Sensor Networks: A Survey of Coordination, Optimization, and Intelligent Approaches

**DOI:** 10.3390/s26217006

**Published:** 2026-11-05

**Authors:** YoungKyun Oh, SeongKeun Jang, BeomKyu Suh, Ki-Il Kim

**Affiliations:** Department of Computer Science and Engineering, Chungnam National University, Daejeon 34134, Republic of Korea

**Keywords:** wireless sensor networks, multiple mobile sinks, data collection, multi-sink coordination, cross-layer optimization, intelligent coordination

## Abstract

Mobile sinks have been widely studied to mitigate the energy-hole problem and improve data collection in wireless sensor networks. Although existing surveys have primarily examined sink mobility, routing, and energy efficiency, they provide limited analysis of the interdependent coordination and optimization problems arising from multiple mobile sinks. To address this gap, this survey presents a coordination-centered review of multiple-mobile-sink-assisted data collection, organizing existing studies into five research dimensions within three complementary analytical levels: decision problems, decision coupling, and solution paradigms. These dimensions encompass task assignment and network partitioning, coordinated trajectory planning, multi-sink routing and data collection, cross-layer coupling and joint optimization, and intelligent multi-sink coordination. Beyond conventional classification, we analyze decision dependencies, distinguish sequentially coupled approaches from explicit joint optimization, and compare representative solution methodologies and their operational trade-offs. We further examine reinforcement learning and multi-agent reinforcement learning alongside alternative learning paradigms, distinguishing learning-based adaptation, federated parameter sharing, and explicit cooperation among multiple physical mobile sinks. We also identify open challenges involving learning scalability and heterogeneous multi-sink coordination, as well as security and practical deployment constraints.

## 1. Introduction

WSNs have been widely deployed in various applications, including industrial automation, environmental and infrastructure monitoring, disaster management, and the Internet of Things (IoT) [1,2,3]. In these applications, a large number of resource-constrained sensor nodes are used to generate sensing data and forward them toward a sink node through single- or multi-hop communication in an ad hoc manner [4].

**Figure 1:**
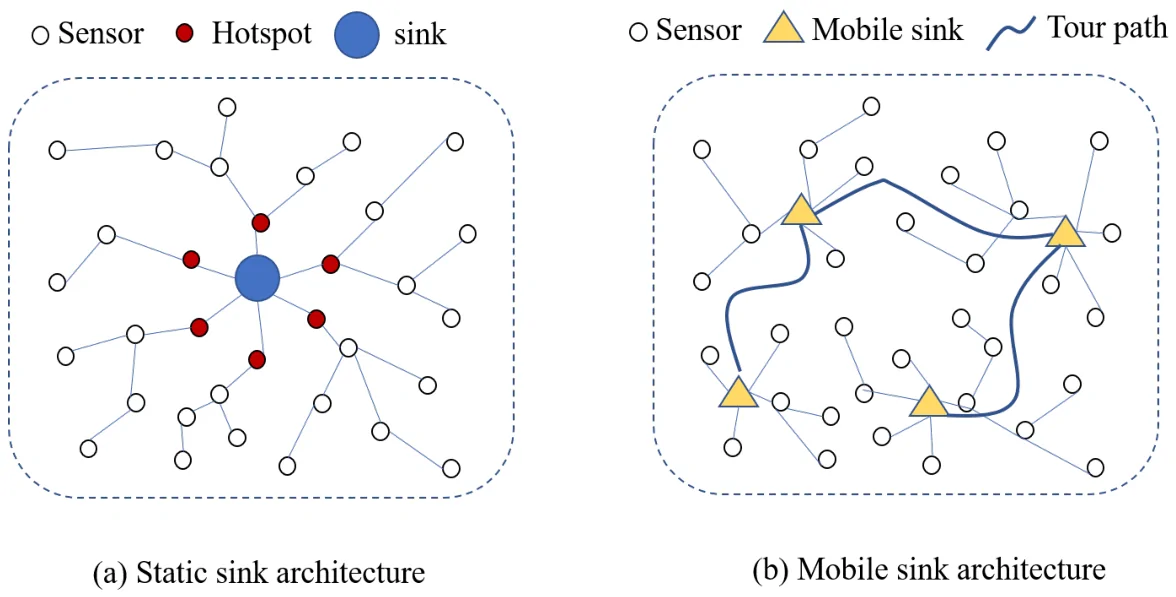
Illustration of the hotspot (energy-hole) problem and its mitigation using mobile sinks. A static sink imposes a persistent relay burden on nearby sensor nodes, causing faster energy depletion. Mobile sinks change the data-collection location over time, reducing persistent multi-hop forwarding and distributing the forwarding load across the network.

In conventional static-sink WSNs, sensor nodes located close to the sink tend to deplete their battery faster than other nodes because they must relay traffic generated by distant nodes in addition to transmitting their own sensing data. This phenomenon, commonly known as the hotspot or energy-hole problem, can cause premature network partitioning even when a substantial amount of residual energy remains in other parts of the network [5,6]. To alleviate this problem, various approaches, including transmission-power control, energy-aware routing, and multiple static-sink deployment, have been investigated. Nevertheless, routing and clustering mechanisms under static-sink deployments may still suffer from spatially uneven energy consumption, particularly around heavily loaded forwarding regions near the sink [7]. Figure 1 illustrates how sink mobility mitigates the hotspot problem by changing the data-collection location and distributing the forwarding burden across different parts of the network.

Mobile sinks can provide an alternative solution by allowing data-collection points to change their positions over time. By moving closer to sensor nodes, cluster heads (CHs), or designated Rendezvous Points (RP), a mobile sink can extend network lifetime by reducing multi-hop forwarding overhead and distributing relay traffic more evenly [8,9]. Sink mobility, however, introduces a new set of interdependent design problems involving trajectory planning, visiting-point selection, sojourn-time control, clustering and routing, data buffering, and communication scheduling [10]. Early research on mobile-sink-assisted WSNs primarily considered a single-mobile-sink, in which one sink moves through the sensing field and collects data either directly from sensor nodes or indirectly through CHs or RPs. Accordingly, extensive research has investigated trajectory planning, rendezvous-point selection, clustering and routing, and energy-aware data-collection strategies to determine how a mobile sink should move and collect data while balancing energy consumption and collection delay [11,12].

Although a single mobile sink can effectively alleviate the hotspot problem to some extent, its scalability has increasingly and consistently emerged as a major challenge as the network size, sensing area, traffic volume, or delay sensitivity increases. A single sink may require a long trajectory to visit geographically distributed collection points, increasing collection latency and information staleness, and may become a data-collection bottleneck under spatially non-uniform or dynamically generated traffic. These limitations have motivated the use of MMS, in which several mobile collectors operate concurrently to provide parallel and spatially distributed data collection [13].

The introduction of MMS, however, does not simply indicate an increase in the number of data collectors; rather, it fundamentally changes the data collection architecture by introducing new challenges related to sink coordination, trajectory planning, and inter-sink interference among individual sinks. More specifically, the sinks must coordinate their trajectories and collection schedules to avoid redundant coverage, satisfy latency requirements, and control communication and mobility costs. Consequently, MMS-assisted data collection introduces tightly coupled design problems involving task assignment, network partitioning, coordinated trajectory planning, cooperative data collection, synchronization, and inter-sink coordination.

Table 1 highlights that the transition from a single mobile sink to MMS is not merely an increase in the number of data collectors. While a single mobile sink mainly introduces mobility and trajectory-design issues, MMSs require task allocation, coordinated mobility, and inter-sink cooperation. These coupled decisions expand the optimization space and motivate cross-layer and intelligent coordination mechanisms. Therefore, MMS-assisted data collection should be viewed as a coordinated multi-agent data-collection problem rather than a straightforward extension of the single-mobile-sink architecture. The benefits of MMSs depend strongly on how collection responsibilities and mobility resources are coordinated [14,15]. Although parallel operation can reduce latency and improve scalability, poor task assignment or independently planned trajectories may cause workload imbalance, redundant mobility, and excessive coordination overhead. These interdependencies have shifted MMS research from independent mobility and routing design toward cross-layer optimization and adaptive coordination, increasingly supported by ML, RL, and MARL. Despite growing interest in MMS-assisted data collection, existing reviews have largely examined multiple sinks within broader mobility-, routing-, or energy-oriented frameworks. Consequently, MMS-specific coordination problems remain fragmented across different research perspectives.

To address this gap, this survey reviews MMS-assisted data collection in WSNs from a coordination-centered perspective. The main contributions are summarized as follows:

- We present the fundamental concepts of MMS-assisted WSNs, including system and communication models, the motivation for multiple mobile sinks, representative mobility models, and performance metrics for evaluating multi-objective trade-offs.
- We compare existing surveys from an MMS-oriented perspective and identify the limitations of mobility-, routing-, and energy-centric classifications in addressing MMS-specific coordination problems.
- We propose a coordination-centered taxonomy covering five research dimensions: task assignment and network partitioning, coordinated trajectory planning, multi-sink routing and data collection, cross-layer coupling and joint optimization, and intelligent multi-sink coordination. These dimensions are organized into three complementary analytical levels: decision problems, decision coupling, and solution paradigms.
- We analyze representative studies within each research dimension, comparing their design principles, coordination mechanisms, and optimization objectives. We further examine decision dependencies, distinguish sequential coupling from explicit joint optimization, and compare solution methodologies in terms of their strengths, limitations, and operational trade-offs.
- We review emerging RL- and MARL-based approaches for adaptive and cooperative multi-sink control. We also examine alternative learning paradigms and distinguish learning-based adaptation, federated parameter sharing, and explicit cooperation among multiple physical mobile sinks.
- We identify open research challenges involving scalable dynamic coordination, coupled multi-objective decision-making, coordination overhead, and heterogeneous multi-sink operation. We further discuss security-aware coordination, practical deployment constraints, and corresponding evaluation requirements.

The remainder of this paper is organized as follows. Section 2 describes the literature search strategy, study selection criteria, and scope of the review. Section 3 introduces the fundamentals of MMS-assisted WSNs, including the system model, mobility models, and performance metrics. Section 4 reviews existing surveys and identifies the research gap motivating this work. Section 5 presents the proposed taxonomy and reviews representative studies on task assignment and network partitioning, coordinated trajectory planning, multi-sink routing and data collection, cross-layer coupling and joint optimization, and intelligent coordination. Section 6 discusses open research challenges and future research directions. Section 7 concludes the paper.

## 2. Literature Search and Selection Methodology

This study adopts a critical survey approach to examine coordination and optimization mechanisms for MMS-assisted data collection in WSNs. The review focuses on representative technical contributions, methodological diversity, and research developments rather than claiming exhaustive systematic coverage.

The literature review covers foundational and recent studies published up to October 2026. The search strategy targets research on MMS-assisted data collection using terms such as “multiple mobile sinks”, “multi-mobile sink”, “multiple mobile collectors”, and “multiple mobile elements”, combined with “wireless sensor networks”, “data collection”, “task assignment”, “trajectory planning”, “routing”, “coordination”, and “joint optimization”. The literature was updated during manuscript revision to incorporate additional foundational and recent studies.

The reviewed publications originate from diverse academic publishers and professional societies, including IEEE, Elsevier, Springer Nature, MDPI, Wiley, and IET. These publication sources represent the bibliographic coverage of the review and should be distinguished from the databases used to identify relevant studies. The bibliographic records alone do not establish which search platforms were used. Core studies were selected when they involved at least two physically mobile data collectors and addressed task assignment and network partitioning, coordinated trajectory planning, multi-sink routing and data collection, cross-layer coupling and joint optimization, or intelligent multi-sink coordination. Foundational studies were retained for their technical contributions, while recent studies were included to examine emerging optimization and learning-based methods.

Studies involving only a single mobile sink, stationary multi-sink deployments, or generic multi-agent methods without a direct data-collection context were excluded from the core analysis. Related studies were considered separately when they provided transferable techniques or relevant application-specific insights. Candidate publications were examined using their titles, abstracts, bibliographic metadata, and available full texts. Duplicate records were identified using publication titles and Digital Object Identifiers (DOIs). The reviewed literature was selected from peer-reviewed scholarly publications, primarily journal articles and conference papers. Preprints available only through repositories such as arXiv were excluded. When both conference and journal versions of a study were available, the journal version was generally preferred. For studies addressing similar research problems, more recent publications were prioritized, provided that they offered relevant methodological developments or additional analytical insights. Earlier studies were retained when necessary to explain foundational concepts or the evolution of MMS research.

Selected studies were classified according to their primary research objectives and technical contributions, with secondary dimensions assigned when additional decision problems or solution methods were involved. The classification distinguishes three complementary analytical levels: decision problems, decision coupling, and solution paradigms. Sequentially coupled methods are distinguished from explicit joint optimization, while learning-based approaches are examined according to whether they support individual decisions or cooperation among multiple physical mobile sinks.

The review considers differences in evaluation objectives, experimental conditions, and performance metrics when analyzing representative studies. Reported improvements are interpreted relative to their original baselines and network configurations. The absence of complete historical search logs, database-specific retrieval counts, and screening records limits the reproducibility of the literature search. This review therefore provides a critical analysis of representative MMS approaches without claiming exhaustive systematic coverage.

## 3. Fundamentals of Multiple-Mobile-Sink-Assisted WSNs

MMS-assisted wireless sensor networks (MMS-WSNs) employ multiple mobile data collectors that operate concurrently within the sensing field. This section presents the fundamental concepts required to understand MMS-assisted data collection. We first describe the system and communication model, followed by the motivation for employing MMS. We then discuss representative mobility models and summarize the principal performance metrics used to evaluate MMS-assisted WSNs.

### 3.1. System and Communication Model

Consider a WSN consisting of a set of stationary sensor nodes $N=\{n_{1},n_{2},\ldots ,n_{N}\}$ deployed over a sensing region A and a set of mobile sinks $M=\{m_{1},m_{2},\ldots ,m_{M}\}$, where M≥2. Sensor nodes periodically or event-drivenly generate sensing data and deliver the generated packets to one of the mobile sinks. Depending on the network architecture, data may be transmitted directly to a nearby mobile sink or forwarded through intermediate sensor nodes, CHs, or RPs. Let $N_{j}\subseteq N$ denote the set of sensor nodes assigned to mobile sink $m_{j}$. Each mobile sink follows a trajectory within the sensing field and collects data while moving or during temporary stops at designated collection locations. Consequently, data delivery in an MMS-WSN depends jointly on the communication topology and sink trajectories. Sensor nodes close to a sink trajectory can often communicate directly with a mobile sink, whereas distant nodes may rely on multi-hop forwarding. The resulting network topology is therefore time-varying even when the sensor nodes themselves remain stationary.

The communication model typically assumes that the energy consumed by a sensor node depends strongly on transmission distance. Introducing multiple mobile sinks can reduce the average sensor-to-sink distance and the number of forwarding hops by allowing sinks to approach different portions of the sensing field simultaneously. Accordingly, MMS-assisted data collection can be viewed as a joint optimization problem involving three closely coupled components: communication, which determines how sensor data are forwarded; mobility, which determines where and when each sink moves; and coordination, which determines how collection responsibilities and mobility resources are distributed among multiple sinks.

The baseline MMS model considered in this survey consists of stationary sensor nodes and multiple physically mobile data collectors. Each mobile sink moves through the sensing field and collects data either directly from sensor nodes or through intermediate forwarding structures. Several reviewed studies extend this baseline model by introducing mobile sensor nodes, UAV-based collectors, or mobile ground platforms. Although these systems share certain data-collection and coordination problems, they differ in mobility constraints, communication characteristics, energy models, and network dynamics. Therefore, their results should not be interpreted as directly comparable under a single homogeneous system model. Furthermore, cooperation among functional learning agents does not necessarily constitute cooperation among multiple physical mobile sinks. Studies employing separate learning agents for clustering, routing, or resource management are considered methodologically related unless they explicitly involve at least two physically mobile data collectors.

### 3.2. Motivation for Multiple Mobile Sinks

The primary motivation for employing multiple mobile sinks is to overcome the scalability and latency limitations of single-mobile-sink-assisted WSNs. As the sensing area, network size, or traffic volume increases, a single mobile sink may require a long collection tour, resulting in increased revisit intervals, collection latency, and information staleness.

MMSs address these limitations through parallel and spatially distributed data collection. Collection responsibilities can be distributed among multiple sinks, with each sink serving a subset of sensor nodes, clusters, RPs, or geographical regions. This can shorten individual collection tours, reduce sensor-to-sink communication distances, and improve scalability. Moreover, collection responsibilities can be dynamically redistributed according to spatially non-uniform traffic, residual energy, or changing network conditions.

These benefits depend on effective coordination among the mobile sinks. Overlapping service regions, workload imbalance, redundant movement, trajectory conflicts, and coordination overhead can offset the advantages of parallel collection. Consequently, the fundamental design problem shifts from optimizing the mobility of an individual sink to coordinating the collective communication and mobility resources of multiple sinks.

### 3.3. Research Trends in Multiple-Mobile-Sink-Assisted Data Collection

Research on MMS-assisted data collection has progressively shifted from fundamental multi-sink coordination and mobility-aware routing toward optimization-based data collection and more integrated approaches to task assignment, trajectory planning, and adaptive coordination.

Early MMS research primarily investigated how multiple mobile sinks could cooperate to improve data-collection efficiency and balance network resource utilization. Chatzigiannakis et al. [16] explored centralized and distributed coordination mechanisms for balancing collection responsibilities and reducing overlapping sink operations. These efforts established the importance of inter-sink coordination, demonstrating that deploying multiple collectors alone does not necessarily guarantee efficient data collection. As multiple sinks move through the sensing field, their changing positions introduce additional challenges for routing and data delivery. Kweon et al. [17] investigated grid-based routing for communication between multiple sources and mobile sinks. Han et al. [18] further considered energy-efficient and low-latency routing-tree construction, while Wang et al. [19] investigated distance-aware routing to reduce energy consumption in networks with multiple mobile sinks. Collectively, these studies highlighted the need to coordinate mobility-dependent routing decisions while balancing energy efficiency, network lifetime, and collection latency.

Subsequent research broadened the focus from routing efficiency toward more sophisticated data-collection and optimization mechanisms. Huang and Lin [20] investigated many-to-many data collection for mobile users, providing a related perspective on multicast and aggregation-based data delivery. Although this architecture is not necessarily equivalent to cooperative MMS operation, it illustrates communication mechanisms relevant to multiple data-collection destinations. Yang et al. [21] further investigated optimization-based routing for multiple mobile sinks using discrete Particle Swarm Optimization. These developments demonstrated that efficient multi-sink operation requires consideration of not only individual routing paths but also the interactions among multiple collection destinations and network resources. These early studies established the foundations of MMS research by addressing multi-sink coordination, mobility-aware routing, and optimization-based data collection, paving the way for more integrated and adaptive approaches.

Recent MMS research continues to develop the links among collection-task allocation, route construction, and mobility control. Ding et al. [22] proposed a dual-clustering-based multi-mobile-sink path-planning approach with an improved arithmetic optimization algorithm, illustrating how partitioning decisions and sink trajectories can be designed together for application-specific collection requirements. In a complementary direction, Xu [23] examined delay-sensitive query processing by combining rendezvous-point selection, the optimization of the number of mobile sinks, and movement-path planning. Together, these studies indicate that recent MMS designs increasingly couple the number and responsibilities of collectors with their routes and the timeliness of data acquisition. Their different settings also caution against directly comparing reported improvements without accounting for sensor density, movement constraints, traffic models, and evaluation baselines.

### 3.4. Mobility Models

Mobility models determine how multiple sinks [24] move through the sensing field and strongly influence communication distance, collection delay, energy consumption, and coordination complexity. In MMS-WSNs, mobility models can be broadly characterized according to the degree of control and coordination among sinks. Table 2 summarizes the main characteristics of representative mobility models for MMS-assisted WSNs. In addition to movement patterns, advantages, and limitations, the comparison explicitly examines how each mobility model influences routing stability, sink discovery, and task assignment. Predetermined mobility generally facilitates stable routing and fixed task allocation, whereas coordinated and adaptive mobility enables dynamic task redistribution at the expense of additional control overhead. Hybrid mobility combines these characteristics by maintaining predictable baseline collection while allowing selected sinks to respond to time-varying sensing demands. Unlike the other mobility categories, hybrid mobility is not defined by a single trajectory generation mechanism. Rather, it represents a composite configuration in which different sinks employ different mobility policies within the same MMS system. Its effectiveness depends on how these policies are coordinated through task allocation, inter-sink information exchange, and collection handover.

- Random Mobility: Under random mobility, each sink moves according to a stochastic rule, such as random walk, random waypoint, or random direction mobility. This approach requires little trajectory-planning overhead and can be appropriate when environmental information is unavailable. However, independent random movement may lead to uneven spatial coverage, redundant visits, and long inter-visit times.
- Predetermined Mobility: In predetermined mobility, each mobile sink follows a predefined route, such as a fixed path, circular trajectory, grid route, or patrol path. The sensing area can also be partitioned into multiple regions, with each sink assigned a predefined route within its own region. It provides predictable contact opportunities and simplifies routing and scheduling. Its main limitation is its limited ability to respond to changes in traffic, residual energy, link quality, or network conditions.
- Controlled and Optimized Mobility: Controlled mobility determines sink trajectories according to network objectives such as energy consumption, tour length, collection delay, or network lifetime. Candidate collection locations may correspond to sensor nodes, CHs, RPs, or optimized sojourn points. For multiple sinks, the problem generally includes both assigning collection locations to individual sinks and determining an efficient trajectory for each sink. This creates a coupled assignment–routing problem that is substantially more complex than single-sink trajectory optimization.
- Coordinated and Adaptive Mobility: More advanced MMS systems coordinate sink trajectories according to time-varying network states. Sink positions, traffic demand, residual energy, queue states, and link conditions can be used to dynamically modify collection responsibilities and movement decisions. Coordination may be centralized, where a controller jointly determines the actions of all sinks, or distributed, where individual sinks make local decisions while exchanging limited information with other sinks. Recent learning-based approaches further extend adaptive mobility by using Reinforcement Learning (RL) or Multi-Agent Reinforcement Learning (MARL). In such systems, each mobile sink can be modeled as an agent whose actions affect not only its own collection performance but also those of other sinks. This makes coordinated learning particularly relevant to dynamic MMS-WSNs, although convergence, communication overhead, scalability, and stability remain important challenges.
- Hybrid Mobility: Hybrid mobility [25,26] combines predictable routes for some sinks with event-triggered or adaptive movement for others. Scheduled collectors handle periodic traffic, while adaptive collectors serve event hotspots or overloaded regions. A hybrid controller requires shared task information, overlap resolution, and handover mechanisms between scheduled and adaptive coverage. Fixed-route sinks support predictable wake-up schedules and stable routing, whereas adaptive sinks require sink-discovery updates or route adjustments. Task assignment must account for contact predictability, demand variation, and reassignment overhead. Hybrid mobility is particularly suitable for networks combining predictable traffic with localized event-driven demand, but its benefits may diminish when coordination overhead exceeds the gains from adaptive movement.

### 3.5. Performance Evaluation of MMS-Assisted WSNs

Performance evaluation of MMS-assisted WSNs involves three closely related aspects: the selection of performance metrics, the assessment of results under different experimental conditions, and the formulation of optimization objectives based on application-specific requirements.

#### 3.5.1. Performance Metrics

The performance of MMS-assisted WSNs cannot be adequately evaluated using a single metric because improvements in one objective may degrade another. For example, increasing sink mobility may reduce sensor communication energy but increase mobility cost, while aggressive parallel collection may reduce delay at the expense of coordination overhead. Consequently, MMS schemes should be evaluated using multiple communication-, mobility-, and coordination-related metrics. These metrics [27] therefore provide the basis for evaluating the multi-objective trade-offs considered in subsequent sections.

- Reliability and Delivery: Packet delivery ratio, packet loss rate, throughput, and connectivity are commonly used to evaluate communication reliability. These metrics are affected by both routing decisions and the temporal availability of mobile sinks.
- Energy Consumption and Network Lifetime: Energy efficiency remains a fundamental metric because sensor nodes are typically battery constrained. Relevant measures include total and average energy consumption, residual-energy distribution, energy consumed per delivered packet, and network lifetime.
- Collection Latency: Collection latency measures the time required for generated data to reach a mobile sink or final destination. Multiple sinks can reduce latency through parallel collection, but the actual improvement depends on sink assignment, trajectories, and scheduling.
- Mobility Cost: Mobility-related metrics include individual and total travel distance, travel time, velocity requirements, number of stops, and physical movement energy. In multiple-sink systems, both the maximum route length of an individual sink and the aggregate movement cost of all sinks are relevant because minimizing total distance alone may produce highly unbalanced sink tasks.

#### 3.5.2. Evaluation Conditions and Performance Assessment

This section examines two aspects of performance evaluation in MMS-assisted WSNs. The first concerns the evaluation conditions required to interpret and compare the results of existing studies. The second addresses how application-specific requirements can be incorporated into optimization objectives and operational constraints. Section 3.5.1 introduces the principal performance metrics for MMS-assisted data collection. The reviewed studies, however, employ different evaluation objectives, network configurations, and experimental conditions, making direct comparisons of reported performance improvements difficult without considering these differences.

The studies in Table 3 evaluate MMS approaches using different performance objectives, including network lifetime, energy consumption, collection latency, coverage, and routing efficiency. The table summarizes their evaluation focus and methodological assumptions rather than establishing direct numerical comparisons. Reported metrics may differ in definition, measurement procedures, and experimental conditions, making performance improvements difficult to compare across studies. These performance objectives often involve trade-offs. Increasing sink mobility may reduce sensor communication energy while increasing travel distance and mobility cost. Deploying additional sinks may reduce collection latency but increase coordination overhead. Reported performance also depends on network size, sensor density, sensing-area dimensions, sink mobility constraints, traffic generation, radio-energy models, and collection schedules. Performance gains should therefore be interpreted relative to the baselines and experimental conditions of each study.

Reliable performance assessment requires clearly defined metrics and consistent evaluation conditions. Energy consumption and network lifetime should be examined alongside collection latency, reliability, mobility cost, and coordination overhead. Numerical comparisons should be restricted to studies with compatible experimental settings or supported by appropriate normalization. This approach provides a more balanced assessment of the benefits and operational costs of MMS coordination.

#### 3.5.3. Application Requirements and Optimization Objectives

The relative importance of performance metrics depends on the application requirements of MMS-assisted WSNs. In periodic environmental monitoring, energy efficiency and network lifetime are often prioritized, provided that collection deadlines are satisfied. In industrial or event-driven monitoring, reliability and worst-case collection latency may take precedence over energy savings. For large-scale or infrastructure-constrained monitoring, mobility cost, connectivity, and coordination overhead become particularly important. Accordingly, metric priorities should be specified for each application rather than assuming a universal optimization objective. Table 4 provides an illustrative comparison.

Application-specific performance requirements can be represented by a weighted objective that minimizes normalized energy consumption, collection latency, mobility cost, and coordination overhead:

(1) $$\min_{M\in M}J\left(M\right)=w_{E}\hat{E}\left(M\right)+w_{L}\hat{L}\left(M\right)+w_{C}\hat{C}\left(M\right)+w_{O}\hat{O}\left(M\right),$$

where M is the set of feasible sink counts, *M* is the number of mobile sinks, and $\hat{E}$, $\hat{L}$, $\hat{C}$, and $\hat{O}$ denote normalized energy consumption, collection latency, mobility cost, and coordination overhead, respectively. The weights satisfy $w_{i}\geq 0$ and $\sum_{i}w_{i}=1$. Strict reliability and deadline requirements are treated as feasibility constraints. The effect of sink count on collection time can be illustrated using an ideal workload-sharing model. For a fixed aggregate travel distance *D* and identical sink velocity *v*, perfectly balanced parallel operation without service or coordination delays gives

(2) $$T_{\mathrm{ideal}}\left(M\right)=\frac{D}{Mv}.$$

Actual completion time also depends on individual routes, service times, and coordination delays. Assuming parallel sink operation and non-overlapping coordination delay, the collection-round completion time is approximated by

(3) $$T_{\mathrm{round}}\left(M\right)=\max_{1\leq j\leq M}\left(\frac{d_{j}\left(M\right)}{v_{j}}+t_{j}^{\mathrm{service}}\left(M\right)\right)+T_{\mathrm{coord}}\left(M\right),$$

where $d_{j}\left(M\right)$, $v_{j}$, and $t_{j}^{\mathrm{service}}\left(M\right)$ denote the trajectory length, velocity, and service time of sink *j*, respectively. $T_{\mathrm{coord}}\left(M\right)$ represents coordination delay outside parallel collection. Additional sinks may shorten individual routes without reducing aggregate mobility cost:

(4) $$C_{\mathrm{mob}}\left(M\right)=\sum_{j=1}^{M}c_{j}d_{j}\left(M\right),$$

where $c_{j}$ denotes the movement cost per unit distance of sink *j*.

Coordination costs also depend on the communication architecture. All-to-all communication requires M(M−1) directed messages per coordination round, whereas star-based coordination typically requires O(M) messages. Communication energy additionally depends on message size and link conditions. These models illustrate why increasing the number of mobile sinks does not necessarily improve overall performance. The benefits should be assessed using consistent network configurations, traffic conditions, mobility constraints, and collection requirements.

## 4. Comparative Analysis of Existing Surveys

Existing surveys of mobile-sink-assisted data collection have emphasized sink mobility, routing, communication mechanisms, and network lifetime. Several also discuss MMSs, but their analytical frameworks differ from the coordination-centered perspective adopted here. This section compares their coverage, identifies limitations relevant to MMS operation, and explains the distinctive contributions of this survey.

### 4.1. Existing Surveys on Mobile-Sink-Assisted WSNs

Table 5 compares representative surveys in terms of MMS coverage and their treatment of partitioning, mobility, routing, optimization, and coordination. The symbols 🗸, Δ, and – indicate substantial coverage, partial coverage, and no substantive treatment identified, respectively. These assessments reflect the emphasis of each survey rather than the mere occurrence of related terms.

Khan et al. [33] classified mobile-sink data-collection schemes by mobility constraints and control, whereas Tunca et al. [34] focused on distributed routing and sink-location management. Nguyen and Nguyen [35] explicitly distinguished multiple mobile sinks within a broader review of mobility-based network-lifetime improvement. Kim et al. [36] examined communication techniques in relation to mobility patterns, and Temene et al. [37] surveyed mobility mechanisms across WSN architectures. These reviews provide important background on mobility and communication without consistently placing inter-sink coordination at the center of their analyses. Ari et al. [38] reviewed IoT data-collection architectures and protocols, while Zafor et al. [39] examined mobile-sink data collection and sensor recharging. These recent surveys extend the context for mobile data gathering but have different principal objectives from a coordination-centered MMS review.

### 4.2. Limitations of Existing Surveys

The main limitation of existing reviews is not an absence of MMS coverage but the limited integration of interdependent decisions involving multiple physical collectors. A sensor-to-sink assignment can change the required trajectory, and that trajectory can affect routing, collection latency, and energy consumption. Mobility- or routing-centered taxonomies do not always expose these dependencies or the costs of coordinating them. A second limitation concerns the distinction between coupling and joint optimization. Partitioning, trajectory construction, routing, and scheduling may be executed sequentially without being optimized jointly. An MMS-centered analysis should distinguish such staged designs from formulations that explicitly couple and optimize multiple decision variables.

Learning-based methods introduce an additional classification challenge. RL or MARL can support a specific decision problem or several coupled problems, and cooperation among functional learning agents does not necessarily represent cooperation among physical mobile sinks. Reviews focused mainly on traditional mobility or routing categories may not make these distinctions explicit. Performance assessment also requires attention to differing network sizes, sink counts, mobility constraints, traffic models, and metric definitions. Improvements in lifetime, latency, or energy consumption cannot be treated as directly comparable across incompatible experimental settings; coordination and movement costs further qualify reported benefits. Table 6 summarizes the primary analytical focus of each survey and the MMS-specific questions that receive less emphasis in its stated scope.

### 4.3. Distinctive Contributions of This Survey

This survey organizes MMS research into five interconnected dimensions across three complementary analytical levels: decision problems, decision coupling, and solution paradigms. The decision problems encompass task assignment and network partitioning, coordinated trajectory planning, and multi-sink routing and data collection. Cross-layer coupling and joint optimization characterize relationships among these decisions, while intelligent multi-sink coordination represents a solution-oriented perspective. Primary and secondary classifications identify each study’s principal contribution and related decision variables without treating overlapping characteristics as mutually exclusive categories. The analysis distinguishes sequential coupling from explicit joint optimization and individual learning-based adaptation from cooperation among multiple physical mobile sinks.

The survey also compares solution methodologies, coordination requirements, and evaluation conditions to explain when reported benefits depend on particular assumptions. These analytical distinctions complement the topic-based coverage of earlier reviews and motivate the taxonomy presented in Section 5.

## 5. Taxonomy of Multiple-Mobile-Sink-Assisted Data Collection

The use of MMSs transforms data collection from an individual mobility problem into a coordinated resource-allocation and control problem. An MMS system must determine how collection responsibilities are distributed, how sinks move, how sensor data are delivered, and how these decisions adapt to changing network conditions.

This survey organizes MMS-assisted data collection into five research dimensions: (1) task assignment and network partitioning, (2) coordinated trajectory planning, (3) multi-sink routing and data collection, (4) cross-layer coupling and joint optimization, and (5) intelligent multi-sink coordination.

**Figure 2:**
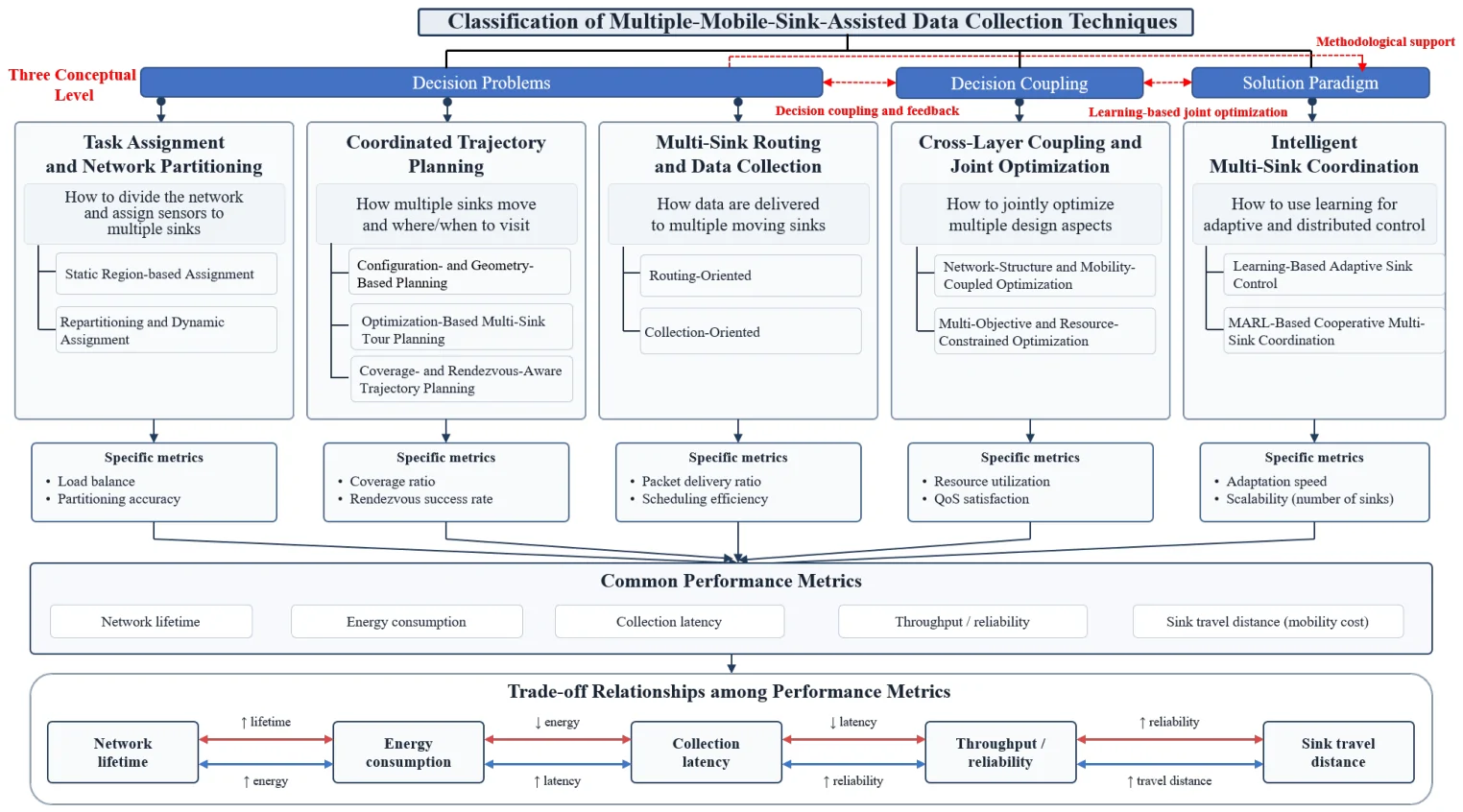
Coordination-centered taxonomy of MMS-assisted data collection across three complementary analytical levels: decision problems, decision coupling, and solution paradigms.

Figure 2 presents these dimensions within three complementary analytical levels. Decision problems encompass assignment, trajectory planning, and routing, while decision coupling describes dependencies among these decisions. Solution paradigms characterize learning-based methods used to optimize or adapt individual and coupled decisions. These levels are complementary rather than sequential or mutually exclusive. Representative studies are classified according to their principal research contributions. The primary dimension (P) identifies the main decision problem, coupling mechanism, or solution paradigm, whereas secondary dimensions (S) indicate supporting components and technical dependencies. Table 7 illustrates this classification using representative MMS studies. D1–D5 denote task assignment and network partitioning, coordinated trajectory planning, multi-sink routing and data collection, cross-layer coupling and joint optimization, and intelligent multi-sink coordination, respectively.

The classification distinguishes decision dependencies from explicit joint optimization. A sequential procedure that determines network partitions before constructing sink trajectories involves coupled decisions but does not necessarily optimize them jointly. D4 is therefore assigned only when the study explicitly addresses cross-layer coupling or joint optimization, with the nature of the coupling identified in the analysis. Keshari et al. [30] are classified under D2 (primary) and D1 (secondary), whereas Liu et al. [32] are classified under D5 (primary) and D2 (secondary). The former is classified under trajectory planning (D2), while the latter is classified under intelligent coordination (D5), with trajectory planning identified as a secondary dimension in both cases where applicable. Primary and secondary dimensions describe the principal contribution and related technical components rather than mutually exclusive research categories. Learning-based coordination may support individual decision problems or cross-layer optimization without necessarily involving cooperation among multiple physical mobile sinks.

### 5.1. Conceptual Levels of the Coordination-Centered Taxonomy

The three analytical levels describe different but interconnected aspects of MMS coordination. Decision problems specify what must be determined, decision coupling describes how these decisions interact, and solution paradigms identify methods for optimizing or adapting them. A study may therefore address several decision problems while employing a particular coupling mechanism or learning-based solution method.

The relationship between decision problems and decision coupling depends on which variables are optimized and how information is exchanged. Sequential approaches determine assignments, trajectories, or routing decisions in separate stages, whereas explicit joint optimization considers multiple decision variables within a shared optimization formulation. The presence of multiple design stages alone does not establish joint optimization. Learning-based coordination can address individual decision problems or their coupled optimization. A learning method is not necessarily evidence of cooperation among multiple physical mobile sinks. The primary and secondary dimensions in Table 7 therefore reflect the main technical contribution and supporting components rather than independent or mutually exclusive research categories.

### 5.2. Task Assignment and Network Partitioning

The fundamental problem in MMS-assisted data collection is how data-collection responsibilities should be distributed among multiple moving collectors. A basic approach to this problem is to partition the sensing field or sensor population and assign each resulting region, group, or collection task to a particular mobile sink. Existing approaches range from static geographical partitioning to adaptive partitioning, cluster-based assignment, and density-aware schemes. We distinguish predefined region-based assignment from configuration-aware and runtime-adaptive assignment approaches. Initial configurations based on node density or network characteristics do not necessarily involve dynamic task reassignment during network operation.

#### 5.2.1. Static Region-Based Assignment

An early and straightforward approach is static region-based assignment. Al-Behadili et al. [40] divided the sensing field into four fixed rectangular regions and assigned one mobile sink to each region. Sensor nodes within each region are organized into clusters, and sensed data are first forwarded to CHs, from which the corresponding mobile sink collects the aggregated data. Each sink operates only within its predefined service region and follows a planned collection path with temporary stopping points for data reception. Such geographical decomposition reduces the complexity and coordination overhead associated with dynamically assigning collection responsibilities. However, fixed partitions cannot readily adapt to changes in traffic distribution, or residual energy.

A more cooperative partition-based approach was proposed by Wen et al. [14]. Their cooperative data collection algorithm partitions *n* static sensor nodes into *k* disjoint groups served by *k* local mobile sinks $m_{1},\ldots ,m_{k}$, while an additional global mobile sink $m_{0}$ collects the aggregated data from the local sinks. For each group, a set of collection points is selected and a local collection path is constructed. The global mobile sink subsequently follows a global path, while rendezvous times and sink speeds are coordinated to enable data transfer from the local sinks to the global sink. This hierarchical architecture combines network partitioning, local data collection, and inter-sink cooperation. The reported evaluation showed improvements in network lifetime, rendezvous time, fairness, and collection efficiency compared with the considered schemes. While such partition-based approaches establish collection responsibilities in advance, adaptive sink coordination becomes important when network conditions vary.

#### 5.2.2. Configuration-Aware and Runtime-Adaptive Assignment

The approaches discussed in this subsection differ in when and how collection responsibilities are determined. Configuration-aware methods use network characteristics, such as node density, residual energy, or spatial distribution, to establish an initial partitioning or sink configuration. In contrast, runtime-adaptive assignment requires collection responsibilities to be updated during network operation in response to changing traffic, connectivity, energy conditions, or sink availability. Therefore, density-aware partitioning, clustering-based configuration, and optimization-based sink placement are not automatically classified as dynamic task reassignment. Such a classification requires explicit evidence of operational reassignment or repartitioning.

Khalid et al. [29] proposed a distributed consensus-based routing protocol in which multiple mobile sinks collaboratively determine sink relocation according to network coverage conditions. The network is partitioned into service regions associated with individual sinks, and relocation decisions are triggered by coverage conditions. Rather than allowing each sink to independently optimize its own movement, participating sinks verify relocation decisions through a distributed consensus mechanism. This approach is intended to prevent an individual relocation decision from adversely affecting the coverage and service responsibilities of neighboring sinks. The scheme therefore represents a transition from predetermined sink responsibilities toward adaptive and collaborative multi-sink control. The deployment of a large number of mobile sinks, however, increases both deployment and maintenance costs. This motivates cluster-based approaches that jointly determine data-collection structures and the required mobile-sink resources.

Srinivas and Amgoth [41] proposed a hierarchical clustering approach for large-scale WSNs in which the number of clusters is not predetermined. Instead, a modified gap statistic is used to determine an appropriate clustering structure, which is subsequently related to the required set of local mobile sinks. Local sinks collect data within their respective clusters according to constructed collection schedules and trajectories, while a global mobile sink gathers data from the local sinks. Khedr et al. [42] further integrated RP assignment with multi-sink path planning. Their FCM-RP scheme employs Fuzzy C-Means clustering to determine rendezvous-point positions and mobile-collector assignments and subsequently applies Ant Colony Optimization (ACO) to construct collection routes. The objective is to select a relatively small number of RPs that allow sensor data to be delivered over short, preferably single-hop, transmissions while simultaneously balancing the path lengths and round-trip times of multiple mobile collectors.

A related optimization-oriented approach was presented by Siddiqui and Chaudhari [43] through the Optimized Multiple Mobile-Sink Energy Efficient Clustering Algorithm (O-MMECA). The method combines energy-efficient clustering, multiple-sink placement, and energy-aware routing and employs an Artificial Bee Colony (ABC)-based optimization procedure. Network characteristics such as energy consumption, cluster stability, and coverage are incorporated into the optimization process. Unlike explicit region-to-sink task-assignment approaches, O-MMECA primarily focuses on configuring clusters and sink positions to improve energy-efficient data collection. Within the proposed taxonomy, O-MMECA is closer to cluster-based multi-sink configuration than to dynamic task assignment. More recently, Verma et al. [44] considered the problem of maintaining balanced collection and mobility costs when sensor nodes are spatially non-uniform. Their framework combines DBSCAN-based density-aware network partitioning, iterative *k*-means-based rendezvous-point selection, and ACO-based mobile-sink path planning. In contrast to fixed geometric partitioning, DBSCAN allows the collection structure to reflect the spatial density of sensor nodes. The subsequent path-planning stage seeks to prevent individual mobile sinks from being assigned disproportionately long collection tours. The reported results indicate a 22.7% reduction in average sink path length relative to the considered FCM-based configuration. Finally, Liu and Guo [45] addressed a more dynamic scenario in which both sensor nodes and multiple sinks are mobile. Their Mobile-Sensor MMS protocol combines hierarchical clustering and routing with coverage-oriented mobile-sink operation. The sensing field is divided into relatively independent virtual units, with one mobile sink responsible for each unit. Mobility- and energy-related factors are considered during clustering, while the resulting CHs construct routing forests rather than forwarding all data directly to a mobile sink. Unlike the baseline MMS model with stationary sensors, this study considers mobile sensor nodes in addition to multiple mobile sinks. Therefore, its mobility assumptions and coordination requirements are not directly equivalent to those of stationary-sensor MMS systems. Among the studies in Table 8, MMSCM is the only scheme that assumes mobile sensor nodes in addition to mobile sinks.

Across these studies, assignment strategies have moved from fixed geographical partitioning toward cluster-, density-, and network-state-aware assignment. Task assignment and trajectory planning are still frequently treated as sequential decisions: the network is first partitioned or collection points are assigned, after which sink trajectories are constructed. Such decomposition may become inefficient under dynamic traffic, heterogeneous sink capabilities, and time-varying network conditions. Table 8 summarizes representative MMS approaches according to their partitioning and assignment strategies, mobility coupling, coordination mechanisms, and optimization objectives. Recent methods further incorporate node density and dynamic network conditions, indicating an increasing coupling between task assignment and other data-collection decisions.

#### 5.2.3. Comparative Analysis of Task Assignment Methods

Static region-based assignment provides predictable collection responsibilities with limited coordination overhead but may lead to workload imbalance under non-uniform or changing traffic conditions. Density-aware partitioning better reflects sensor distribution but does not necessarily support runtime reassignment. Adaptive assignment can redistribute collection tasks according to network conditions, although frequent updates increase signaling and coordination costs. The choice of assignment method therefore depends on traffic variability, workload balance, sink mobility constraints, and reassignment overhead.

### 5.3. Coordinated Trajectory Planning

Once collection responsibilities have been determined, each mobile sink must follow a trajectory that allows it to efficiently serve its assigned nodes or collection points. In MMS systems, however, trajectory planning cannot always be performed independently because the trajectory of one sink can affect the coverage, communication opportunities, and movement decisions of other sinks.

The simplest strategy assigns an independent trajectory to each sink, typically within a predetermined service region. Such an approach reduces coordination complexity and allows conventional single-sink trajectory algorithms to be applied locally. However, independent planning may result in overlapping trajectories, uneven route lengths, redundant coverage, and inefficient use of the available mobile collectors. Coordinated trajectory planning instead considers the trajectories of multiple sinks jointly.

Existing studies on coordinated trajectory planning for multiple mobile sinks can be classified according to how sink trajectories are constructed. From this perspective, we categorize existing approaches into three groups: Configuration- and Geometry-Based Planning, Optimization-Based Multi-Sink Tour Planning, and Coverage- and Rendezvous-Aware Trajectory Planning.

#### 5.3.1. Configuration- and Geometry-Based Planning

Configuration- and geometry-based approaches determine how multiple mobile sinks should move according to predefined spatial configurations or geometric structures. An early representative study was presented by Basagni et al. [28], who considered multiple sinks as a coordinated mobility system rather than independently moving data collectors. Their objective was therefore not simply to minimize the individual travel distance of each sink but to control the locations and movements of multiple sinks to maximize network lifetime. The network is assumed to contain a set of candidate sites at which sinks can be located, and the simultaneous locations of multiple sinks at a given time are represented as a sink configuration. Consequently, trajectory planning is transformed into determining a sequence of multiple-sink configurations and the duration for which each configuration should be maintained. Linear Programming (LP) is employed to establish an upper bound on the achievable network lifetime. The authors further propose a polynomial-time centralized heuristic to transform the LP solution into practical sink movements. Since such a centralized approach requires global network information, a deployable distributed heuristic is also introduced. This study can therefore be regarded as an early form of discrete coordinated mobility rather than continuous trajectory optimization.

**Figure 3:**
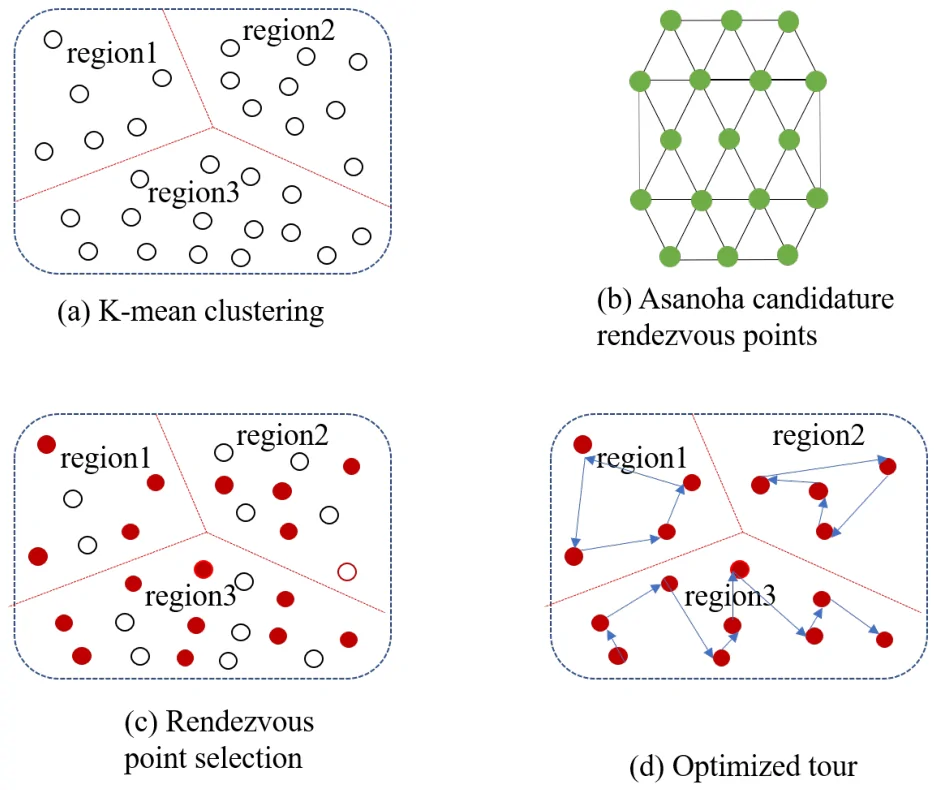
Illustration of the Asanoha-based path-planning procedure for multiple mobile sinks. The procedure consists of network partitioning, generation of candidate rendezvous points (RPs), RP selection and assignment, and construction of optimized multi-sink trajectories. Red lines indicate partition boundaries, green circles represent candidate RPs, filled red circles denote selected RPs, and blue arrows indicate the visiting order and movement direction of the mobile sinks.

More recently, Keshari et al. [46] proposed a geometry-inspired approach for constructing trajectories of multiple mobile sinks. The target region is first partitioned into multiple sub-regions using K-means clustering, with each mobile sink assigned to a corresponding sub-region. Within each sub-region, an Asanoha geometric pattern is employed to generate regularly distributed candidate rendezvous points (RPs) at which a sink can collect sensor data. Rather than requiring the sink to visit every candidate RP, the approach evaluates the generated locations according to multiple criteria and selects an appropriate subset of RPs, thereby eliminating unnecessary collection points. The selected RPs are subsequently used to construct sink-specific trajectories. Compared with earlier configuration- or orbit-based approaches, this method combines spatial partitioning with a predefined geometric pattern to systematically generate collection locations and trajectories. Figure 3 illustrates the main procedure of the Asanoha-based path-planning approach for multiple mobile sinks. First, the sensor network is partitioned into multiple regions, and each region is associated with a mobile sink. Candidate RPs are then generated based on the Asanoha-inspired geometric structure. From these candidates, appropriate RPs are selected and assigned to the corresponding mobile sinks according to the considered optimization criteria. Finally, an individual collection trajectory is constructed for each mobile sink to visit its assigned RPs.

#### 5.3.2. Optimization-Based Multi-Sink Tour Planning

The second category formulates trajectory construction primarily as an optimization problem in which the visiting order, collection locations, and/or multiple sink tours are determined to satisfy network objectives.

Sun et al. [47] proposed an energy-aware path-planning method for multiple mobile sinks with the objective of balancing sensor energy consumption and extending network lifetime. The sensing area is divided into regular hexagonal virtual grids, and candidate parking positions are defined within each grid. Consequently, the original network objective is mapped to determining suitable collection positions, their visiting order, and the allocation of the resulting path among multiple mobile sinks. A Hybrid Positive and Negative Particle Swarm Optimization (HPNPSO) algorithm is employed to solve the resulting path-planning problem, which is formulated as a variant of the selective Traveling Salesman Problem (TSP).

Anwit et al. [48] addressed multi-sink trajectory construction through rendezvous-point selection and multi-tour optimization. Instead of directly constructing sink paths, the approach first determines an appropriate set of RPs for data collection. The selection of a minimum set of RPs is modeled as a set-covering problem and formulated using Integer Linear Programming (ILP). Once the RPs have been determined, the second stage determines the required number of mobile sinks and constructs their corresponding subtours. This multi-tour problem is formulated as a nonlinear optimization problem and addressed using Shark Smell Optimization (SSO). As a result, a single long collection tour is decomposed into multiple subtours assigned to different mobile sinks. The coordination is primarily centralized and offline rather than based on real-time cooperative trajectory adaptation among the sinks. Kumar et al. [13] investigated energy-aware mobile data collection by integrating anchor-point selection, clustering, and path optimization. Their framework employs a modified weighted set-cover approach to determine collection locations, followed by fuzzy C-means clustering and traffic load balancing. Collector routes are subsequently constructed using graph-theoretic techniques based on minimum spanning trees and perfect matching. Unlike approaches that optimize visiting sequences for a fixed set of collection points, this method also considers the number of required mobile data collectors and their travel distances. However, the sequential decomposition of collection-point selection and route construction may limit adaptation to dynamically changing traffic and collection requirements.

Al-Kaseem et al. [49] proposed an energy-efficient path-planning framework integrating region partitioning, clustering, sojourn-location determination, and sink trajectory optimization. First, the sensing field is partitioned according to the number of deployed mobile sinks, with each sink assigned to a corresponding region. Second, a Stable Election Algorithm (SEA) is employed to reduce frequent changes in cluster-head selection and the associated communication overhead. Third, suitable Sojourn Locations (SLs), at which mobile sinks temporarily stop to collect data, are determined through a graph-optimization approach based on the Minimum Weighted Vertex Cover Problem (MWVCP). Finally, the visiting order of the selected SLs within each region is optimized using multiple metaheuristic optimization algorithms, including Genetic Algorithm (GA), ACO, and Simulated Annealing (SA). Because each sink operates within its own region, visiting orders are optimized separately for each region, and coordination among sinks is limited to the initial partitioning.

#### 5.3.3. Coverage- and Rendezvous-Aware Trajectory Planning

Unlike approaches that primarily seek short or energy-efficient tours, coverage- and rendezvous-aware trajectory planning first considers where mobile sinks should collect data and subsequently determines how multiple sinks should visit the selected locations. Wang et al. [50] proposed a representative coverage-aware multi-path scheduling scheme in which parking positions providing high sensor coverage are first identified and then connected through multiple mobile-sink trajectories. An improved Particle Swarm Optimization (PSO) algorithm with a mutation operator is employed to identify parking positions that can cover a large number of sensor nodes. After the parking positions have been determined, a GA is used to schedule multiple trajectories through these locations. Because parking positions are fixed before scheduling, trajectory construction is driven primarily by the quality of collection locations rather than travel distance alone, which distinguishes the method from shortest-tour approaches.

#### 5.3.4. Comparative Analysis of Trajectory Planning Methods

Configuration- and geometry-based approaches provide predictable sink trajectories with relatively low computational complexity. These methods are suitable for networks with stable sensor distributions and collection requirements, but fixed spatial configurations may result in unbalanced travel distances when traffic demands change. Optimization-based approaches determine collection locations and visiting sequences to reduce travel distance, energy consumption, or collection latency. Global optimization can produce efficient routes under well-defined network conditions, although computational complexity and solution time may limit its applicability to dynamic environments.

Coverage- and rendezvous-aware approaches prioritize the selection of collection points that serve multiple sensor nodes. Reducing the number of rendezvous points can shorten sink trajectories but may increase multi-hop forwarding distances and sensor energy consumption. Their effectiveness therefore depends on the balance between collection coverage and communication costs. These approaches also differ in how task assignment and trajectory planning are coupled. Many methods first partition the network or select collection points and subsequently construct sink trajectories. Such sequential designs establish dependencies between decisions but do not necessarily optimize them jointly. Explicit joint optimization requires the assignment and trajectory variables to be considered within a shared optimization formulation or an iterative procedure with clearly defined coupling and convergence conditions.

The selection of a trajectory planning method depends on network stability, traffic variability, collection deadlines, computational resources, and mobility constraints. Reported reductions in trajectory length should be assessed alongside sensor forwarding energy, collection latency, workload balance, and optimization overhead.

### 5.4. Multi-Sink Routing and Data Collection

Mobility and routing are intrinsically coupled in MMS-assisted WSNs. As mobile sinks change their positions, the preferred destination and forwarding path of a sensor node may also change. Routing protocols must therefore determine not only how packets are forwarded but also which mobile sink should receive them. Multi-sink routing introduces the additional problem of sink selection. A sensor may select a sink according to hop count, geographical distance, expected contact time, residual energy, queue state, or estimated delivery cost. Frequent changes in sink position can, however, cause excessive route reconstruction and control-message overhead. Efficient protocols therefore seek to balance route adaptability against routing stability. In this study, existing studies are categorized into Routing-Oriented Approaches and Collection-Oriented Approaches, and the key characteristics and design principles of each category are systematically reviewed.

#### 5.4.1. Routing-Oriented Approach

Routing-oriented approaches focus on how data generated by sensor nodes are efficiently forwarded toward appropriate mobile sinks through routing and forwarding protocols. Unlike task-assignment approaches that primarily determine which sink is responsible for a particular region or set of sensor nodes, routing-oriented approaches focus on establishing energy-efficient and reliable data-delivery paths toward multiple mobile sinks under dynamically changing network conditions.

Gupta et al. [51] addressed hotspot mitigation in heterogeneous WSNs by combining K-medoids clustering with Adaptive Sunflower Optimization for CH selection and a hybrid Harris Hawks Optimization–Ant Colony Optimization (HHO–ACO) scheme for mobile-sink mobility and data collection. Their approach primarily coordinates clustering and sink mobility to distribute the forwarding load and reduce energy depletion around hotspot regions. In contrast, Singh and Malik [52] further couple adaptive sink placement with explicit energy-aware routing. Their approach employs K-medoids clustering and Sooty Tern Optimization for CH selection, Harris Hawks Optimization (HHO) for adaptive mobile-sink placement, and Grey Wolf Optimization (GWO) for energy-efficient routing. Thus, whereas Gupta et al. primarily integrate clustering with mobility optimization for hotspot mitigation, Singh and Malik adopt a more tightly coupled placement–routing design to balance network load, mitigate hotspots, and extend network lifetime.

Another important class of routing-oriented approaches employs clustering to reduce the forwarding burden of sensor nodes. Moussa et al. [53] proposed the Enhanced ACO-based Routing Protocol (EARP), which particularly addresses data-delivery failures caused by faults at critical collection points, such as CHs, or along routing paths. Unlike conventional ACO-based routing approaches that primarily focus on energy efficiency, EARP incorporates path reliability and fault tolerance into route construction. When a failure occurs along an active path, an alternative route can be employed to maintain reliable data delivery. The authors further demonstrated the applicability of the proposed scheme to forest-fire detection, where reliable and timely data delivery is particularly important.

**Figure 4:**
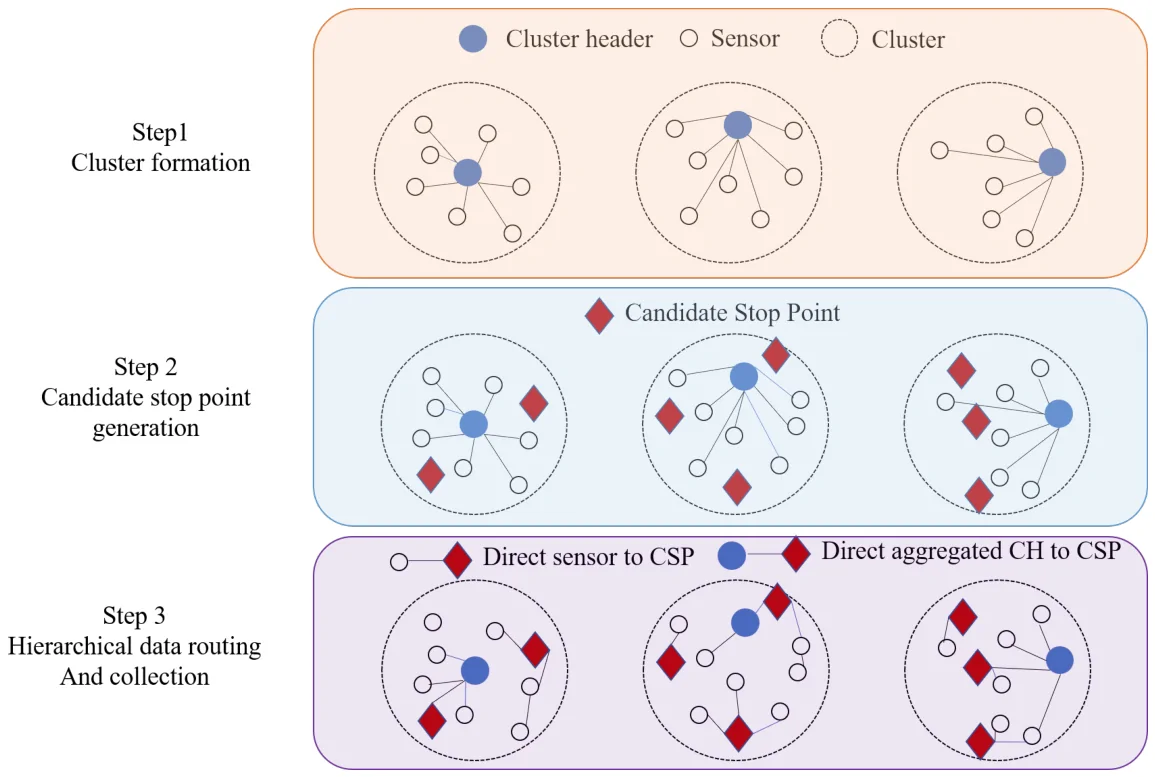
Hierarchical QoS-aware data collection using multiple mobile sinks, where clustered sensing and mobile-sink collection jointly reduce forwarding overhead while supporting energy, latency, and reliability requirements.

Ben Yagouta et al. [54] further investigated hierarchical multi-sink data collection from a QoS perspective. Their approach combines cluster-based hierarchical routing with multiple mobile sinks and evaluates the effects of sink mobility on both energy efficiency and QoS. In particular, the study considers energy consumption, latency, reliability, and throughput, thereby extending the evaluation of multi-sink routing beyond the conventional emphasis on network lifetime. Figure 4 illustrates a QoS-aware data-collection process using multiple mobile sinks. Sensor nodes are first organized into clusters, where cluster heads (CHs) aggregate data from their member nodes. Multiple mobile sinks then move through the network and collect the aggregated data from reachable CHs. This hierarchical collection process reduces excessive multi-hop forwarding while supporting QoS-aware operation in terms of collection delay, reliability, energy consumption, and network lifetime.

Unlike the aforementioned approaches that primarily improve energy efficiency through clustering and hotspot mitigation, Qureshi et al. [55] focused on reducing redundant data transmissions caused by maintaining separate routing paths to multiple mobile sinks. They proposed Combined Path Data Routing, in which a shared forwarding path is divided into multiple sub-branches at selected branching points. A Diagonal Line (DL)-based structure and an opportunistic forwarding metric are further employed to efficiently disseminate data toward multiple mobile sinks. Building upon this shared-path routing concept, Wang et al. [31] proposed Merged Path, a distributed data-dissemination protocol that allows multiple sink-destined routes to share common forwarding segments. Instead of constructing an independent routing path for each mobile sink, Merged Path shares common forwarding paths and creates multiple branches at selected bifurcation points. The protocol employs Discrete Cumulative Clustering (DCC) to group mobile sinks, a Diagonal Virtual Line (DVL) structure to support hierarchical forwarding, and an opportunistic metric to determine data-forwarding decisions. By reducing redundant transmissions associated with separate sink-specific paths, the approach significantly improves energy efficiency in MMS data dissemination.

**Figure 5:**
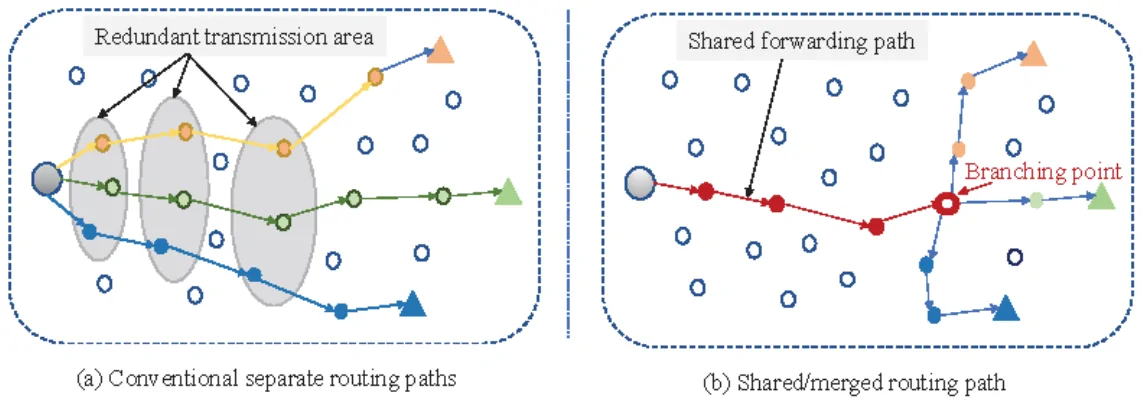
Conceptual comparison of separate and shared routing paths for multiple mobile sinks. (**a**) Conventional sink-specific routing constructs independent delivery paths toward individual mobile sinks, which may result in redundant transmissions over overlapping regions. (**b**) Shared-path routing exploits common forwarding segments and divides the route at selected branching (bifurcation) points to efficiently disseminate data toward multiple mobile sinks, thereby reducing forwarding overhead and energy consumption. Different colors distinguish sink-specific forwarding paths, while the red path in (**b**) represents the shared forwarding segment.

Figure 5 illustrates the fundamental difference between conventional sink-specific routing and shared-path-based multi-sink data dissemination. Instead of maintaining independent routes for individual mobile sinks, shared-path approaches exploit common forwarding segments and branch only when necessary, thereby reducing redundant transmissions and associated energy consumption.

#### 5.4.2. Collection-Oriented Multi-Sink Data Collection

Collection-oriented approaches focus on the mechanisms by which sensed or aggregated data are made available to and acquired by multiple mobile sinks, rather than on the optimization of the sink trajectories themselves.

The first representative study from this perspective was presented by Konstantopoulos et al. [56], who investigated delay-constrained data gathering using multiple mobile elements. Their approach aims to maximize network lifetime while satisfying a given data-gathering delay constraint. To reduce the forwarding burden on energy-constrained sensor nodes, sensors located in energy-rich neighborhoods are selected as rendezvous nodes (RNs) based on residual energy and the energy conditions of their neighborhoods. Two data-gathering protocols, namely cluster-based and convex-hull-based protocols, were proposed. In the cluster-based approach, the network is partitioned into clusters and routing structures are established toward the selected RNs, where sensed data are buffered until they are collected by mobile elements. In the convex-hull-based approach, initial tours are constructed using geometric information and subsequently adjusted by considering the available energy of sensor nodes and the spatial distribution of network energy consumption. Unlike the routing-oriented schemes above, the key design variable here is where data are buffered until collection rather than how packets are forwarded.

Li et al. [57] considered a different data-collection scenario in which sensed data must ultimately be delivered from the WSN to the Cloud. Their work addresses the potential bottleneck associated with uploading a large amount of sensed data to the Cloud by employing multiple mobile sinks as intermediate data collectors. The primary objective is to reduce the energy consumption of sensor nodes while ensuring that data uploading is completed within a specified latency constraint. To this end, the authors proposed an efficient scheduling algorithm that coordinates multiple mobile sinks so that the collected data can be uploaded within the required latency. This work extends the role of multiple mobile sinks from local WSN data collection to latency-constrained sensor-to-Cloud data delivery. Extending this sensor-to-Cloud collection architecture, Wang et al. [58] employed multiple mobile sinks as mobile fog elements and introduced an intermediate fog layer between the WSN and the Cloud. The objective is to increase the overall data throughput while reducing WSN-to-Cloud transmission latency. To support efficient data collection, the authors proposed a Data Collection Fog (DCF) data-collection scheduling algorithm for coordinating the mobile fog elements. In addition, the sensing field is divided into collection zones based on the locations of the mobile sinks using a Voronoi diagram. An energy-efficient routing algorithm is then employed within each collection zone to deliver sensor data to the corresponding mobile sink.

In contrast to approaches employing conventional ground-based mobile sinks, Olivieri de Souza and Endler [59] investigated the use of multiple Unmanned Aerial Vehicles (UAVs) as mobile data collectors. They employed the Distributed Algorithm for Aerial Data Collection (DADCA), which enables multiple UAVs to coordinate their data-collection task through distributed information exchange without relying on continuous centralized coordination. When two UAVs come within communication range, they exchange collection-related information and adjust their cluster-head visitation and collection responsibilities accordingly. The approach also accommodates dynamic changes in the number of available mobile collectors, such as UAV failures or the introduction of additional UAVs, by redistributing collection responsibilities among the active collectors. This local-exchange model removes the need for a central controller, although coordination quality depends on how frequently UAVs encounter one another. Taken together, collection-oriented MMS approaches have progressed from delay-constrained rendezvous-based collection toward scheduled, cooperative, and distributed data-collection architectures. While early approaches primarily coordinated sensor forwarding and mobile-element visits under energy and delay constraints, subsequent studies extended multiple mobile sinks to sensor-to-Cloud and fog-assisted collection scenarios. More recent UAV-based approaches further emphasize distributed coordination and adaptive task redistribution among multiple collectors. This coupling motivates the need for cross-layer MMS frameworks that jointly consider collection efficiency, energy consumption, latency, and coordination overhead.

#### 5.4.3. Comparative Analysis of Routing and Data Collection Methods

Table 9 compares representative routing-oriented and collection-oriented approaches in terms of their principal mechanisms, coordination strategies, system models, and design limitations.

Routing-oriented approaches primarily address forwarding efficiency, route reliability, and multi-sink path selection. In contrast, collection-oriented approaches emphasize data aggregation, rendezvous scheduling, and coordinated data acquisition.

These approaches differ in their network models, delivery objectives, and mobility assumptions. Their reported improvements should therefore be interpreted relative to the original experimental conditions rather than compared as direct numerical rankings. Routing methods are generally more sensitive to sink-location updates and forwarding overhead, whereas collection methods depend more strongly on contact opportunities, buffering requirements, and collection schedules. The appropriate method depends on traffic characteristics, sink mobility, reliability requirements, and collection deadlines.

### 5.5. Cross-Layer Coupling and Joint Optimization

Cross-layer MMS design connects network organization, data forwarding, and mobile-sink operation. These connections can be implemented through sequential decision stages, repeated updates, or a shared optimization formulation. The presence of several design stages alone does not establish joint optimization. This section examines two research themes: network-structure and mobility coupling, and multi-objective optimization under resource constraints. It then discusses decision coupling and compares representative solution methods.

#### 5.5.1. Network-Structure and Mobility-Coupled Optimization

Shi et al. [60] developed an energy-management framework that connects multiple-sink tours, sojourn decisions, and sensor energy consumption. The Chinese Postman Problem is first used to construct sink tours of similar length. Routing trees and energy-aware sojourn locations are then determined using residual-energy information. Because the tours are fixed before subsequent decisions are made, the framework exhibits sequential coupling rather than full joint optimization of mobility and routing.

Vijayashree and Ghana Dhas [61] combined energy-balanced clustering with Artificial Bee Colony (ABC)-based path optimization. Cluster-head (CH) selection responds to residual energy, while the resulting cluster structure guides mobile-sink movement. The approach connects network organization with mobility, but the reported design does not by itself establish simultaneous optimization of all decision variables. Krishnan et al. [62] integrated modified LEACH-based clustering and Ant Colony Optimization (ACO) for multi-sink collection routes. Residual energy influences CH selection; the selected CHs are subsequently assigned to disjoint sink groups, and ACO constructs a route for each group. Clustering, assignment, and routing are therefore linked through a staged procedure, with earlier decisions fixed during route optimization. Mekala and Chatrapati [63] investigated multi-mobile-sink clustering and routing in heterogeneous WSNs using a multi-objective Crow Search Algorithm. The proposed framework connects CH selection and mobile-sink path optimization to improve energy efficiency and data collection. Its degree of joint coupling should be assessed from the shared decision variables, update rules, and constraints rather than inferred solely from the use of a multi-objective metaheuristic.

These studies demonstrate that network-side and mobility-side coordination can be achieved without simultaneously optimizing every decision. Sequential designs simplify individual optimization stages, but fixed upstream decisions may limit subsequent route and sojourn choices.

#### 5.5.2. Multi-Objective and Resource-Constrained Optimization

Poe et al. [64] formulated an Optimal Sink Trajectory problem addressing network lifetime and message-transfer delay. Their formulation considers sink placement, sensor-to-sink assignment, and routing while using sensor-to-sink distance to help manage computational complexity. It illustrates the need to consider energy-related objectives together with delay requirements.

Chen et al. [65] proposed the Lifetime Optimization Algorithm with Multiple Mobile Sink Nodes (LOA_MMSN). Sink paths are constructed before network lifetime is optimized under energy and communication constraints. Because the second stage does not revise the previously selected paths, this method represents sequential cross-layer coupling rather than fully joint trajectory and lifetime optimization. Liang and Luo [66] addressed lifetime maximization subject to an *h*-hop communication constraint and sink travel-distance limits. Their model explicitly links delivery requirements to mobile-sink trajectories and sojourn times. Keskin and Yiğit [67] subsequently incorporated nonzero sink travel times and data generated during sink movement. Their analysis emphasizes that neglecting movement time can distort lifetime estimates, particularly for slow-moving collectors.

These formulations progressively incorporate delivery deadlines, energy budgets, and realistic mobility constraints. Their objectives and feasible solutions differ, however, so improvements reported under different traffic, mobility, and network models should not be treated as directly comparable numerical results.

#### 5.5.3. Decision Coupling and Computational Challenges

Cross-layer optimization in MMS-assisted WSNs involves interdependent decisions concerning task assignment, sink trajectories, routing, and network organization. The extent of decision coupling depends on whether these variables are optimized independently, sequentially, or within a common optimization framework. Sequential approaches determine one set of decisions before optimizing subsequent variables. This decomposition reduces computational complexity but may restrict solution quality because earlier decisions remain fixed. In contrast, explicit joint optimization considers multiple decision variables through a shared objective function or coupled constraints.

Alternating optimization repeatedly updates different variable groups while accounting for their mutual dependencies. Although this approach can reduce the complexity of individual optimization steps, convergence depends on the objective structure and update rules. Global optimality cannot generally be guaranteed for nonconvex MMS problems. Cross-layer optimization also introduces computational challenges associated with discrete assignment and routing decisions, nonlinear mobility constraints, and competing performance objectives. The effectiveness of these approaches should therefore be evaluated in terms of solution quality, computational cost, convergence behavior, and coordination overhead.

#### 5.5.4. Comparative Analysis of Cross-Layer Optimization Methods

Cross-layer methods differ along several methodological axes. Centralized and distributed designs describe where decisions are made; exact, heuristic, and metaheuristic approaches describe how solutions are computed; and static or adaptive operation describes when decisions are updated. These characteristics can coexist within the same method and should not be interpreted as mutually exclusive categories. Table 10 summarizes their operational requirements and limitations.

Centralized coordination facilitates system-level objectives when reliable network-state information is available, whereas distributed coordination favors local autonomy at the cost of information exchange. Heuristic and metaheuristic searches can address complex assignment and trajectory problems without guaranteed global optimality. Adaptive operation is useful when changes in traffic or connectivity justify repeated decision updates. Because these characteristics overlap, method selection should reflect deployment constraints rather than a single method label. Reported energy or latency improvements are meaningful only when considered alongside computation time, mobility costs, signaling requirements, and the conditions under which decisions are updated.

### 5.6. Intelligent Multi-Sink Coordination

MMS-assisted WSNs require sink assignment, movement, and data-collection decisions under changing traffic, energy, and connectivity conditions. Conventional optimization can address these decisions using explicit models and constraints, but repeated computation may be costly when network states change frequently. Learning-based methods can instead adapt selected decisions using observed network states and past experience. Their contribution depends on the decisions being learned and, in particular, whether the learning agents represent distinct physical mobile sinks.

This subsection distinguishes learning-based adaptive sink control from MARL-based cooperative multi-sink coordination. The distinction concerns the scope of the learned decisions rather than the mere use of multiple learning agents. Related learning paradigms are then examined as supporting methods for prediction, network organization, and distributed training.

#### 5.6.1. Learning-Based Adaptive Sink Control

Learning-based adaptive control modifies a particular assignment, collection, or mobility decision in response to network observations. Learning may be performed by a centralized controller or by an individual agent; neither arrangement necessarily entails learned cooperation among physical sinks.

Cheng and Chang [68] addressed the Early Isolation of Specific Sink (EISS) problem through Q-learning-based Adaptive Zone Partition (QAZP). Instead of relying on nearest-sink association, QAZP adjusts collection-zone boundaries according to the residual energy of hotspot nodes. A centralized Mobile Anchor (MA) learns zone-control actions to redistribute forwarding loads across sinks. The approach supports adaptive assignment, but the learning agents do not represent a decentralized team of physical sinks. Senthil Kumar and Prasanth [69] combined QAZP with weighted rendezvous-point (RP) selection and Particle Swarm Optimization (PSO) for collection-route construction. The method first adapts collection zones, selects RPs using factors including hop distance and traffic, and then determines sink paths under a delay bound. Its components form a staged decision process; the use of Q-learning in partitioning does not establish joint learning of assignment and trajectory variables. Vahabi et al. [24] proposed Reinforcement Learning Movement Path (RLMP), which adapts sink movement according to the spatial and temporal distribution of sensing data. The approach targets data-rich collection regions rather than following fixed or random movements and evaluates delivery, delay, lifetime, and energy-related performance. This study illustrates learning applied directly to mobility control, whereas the preceding approaches emphasize adaptive partitioning or combine learned decisions with separate optimization stages.

#### 5.6.2. MARL-Based Cooperative Multi-Sink Coordination

MARL-based coordination models the decisions of multiple agents as interdependent. For direct MMS cooperation, each relevant agent must correspond to a physical mobile sink, and the formulation must specify how sink actions interact through collection responsibilities, travel constraints, shared resources, or common performance objectives. Centralized Training and Decentralized Execution (CTDE) is one possible architecture: global information can support policy training while individual sinks act using available observations during deployment.

**Figure 6:**
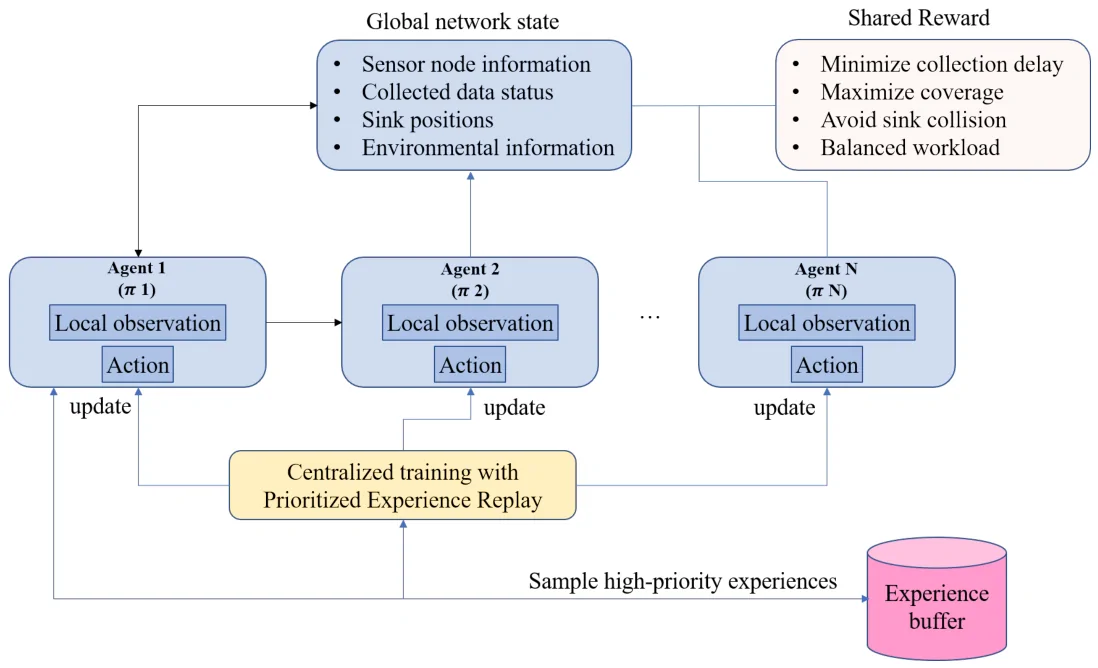
Illustration of MARL-based cooperative trajectory planning for multiple mobile sinks. Individual sink agents select movement actions using available observations, while centralized training uses broader network information and experience replay to update policies. Cooperative objectives can include collection delay and collision avoidance.

Liu et al. [32] considered cooperative path planning for multiple mobile sinks collecting data from cluster heads. Their Multi-Agent Deep Deterministic Policy Gradient (MADDPG) approach models individual sinks as agents and uses combined prioritized experience replay (CPER) to support policy training. The learning objective addresses collection delay and collision avoidance, making cooperation among physical collectors a central part of the method. Figure 6 illustrates the relationship between per-sink decision-making and centralized training. Not all multi-agent learning methods establish physical inter-sink cooperation. Rashid [70] considered energy-harvesting-aware clustering and sink routing using two functional Q-learning agents. One agent selects cluster heads, whereas the other controls routing or sink-movement decisions. The agents represent network functions rather than distinct mobile collectors; thus, this work is relevant to learning-based decision coupling but is not direct evidence of cooperation among multiple physical sinks.

Related UAV-based collection studies illustrate additional cooperative learning architectures. Seong et al. [71] investigated multi-UAV trajectory control for data harvesting and charging using multi-agent deep reinforcement learning, a decentralized partially observable formulation, and Double Deep Q-Networks. Their model accounts for navigation and collision constraints. Its aerial mobility and energy assumptions should be distinguished from those of ground-based MMS-WSNs when interpreting performance. Gao et al. [72] studied UAV trajectory planning and ground-device communication scheduling using Multi-Agent Federated Reinforcement Learning (MAFRL). UAVs learn locally and exchange model updates for aggregation while optimizing collection-related actions under mobility and communication constraints. Federated aggregation describes how policies are trained and shared; it does not by itself prove coordinated task allocation or movement. The operational coordination mechanism must be assessed separately from the federated learning architecture.

#### 5.6.3. Alternative Learning Paradigms for Multi-Sink Coordination

RL and MARL support sequential and interdependent decision-making, but other learning paradigms can assist MMS coordination without directly generating sink-control policies. Supervised learning can predict traffic, link quality, or energy demand for anticipatory scheduling. Unsupervised methods can organize sensor groups and candidate collection regions, although improved clustering quality does not automatically imply lower collection latency or energy consumption.

Juwaied and Jackowska-Strumillo [73] investigated DEC-KM, combining K-means clustering with an energy-efficient clustering protocol. Related KNN-based clustering and comparative analyses were reported by Juwaied et al. [74,75]. These studies offer potentially transferable approaches to clustering and energy-aware network organization; they should not be classified as direct MMS coordination studies unless multiple physical mobile collectors are explicitly modeled. Graph neural networks can represent network topology and neighborhood relationships for sink assignment or route selection, while federated learning can support model training across distributed collectors without centralizing all raw observations. Their practical value depends on inference cost, graph maintenance, heterogeneous local data, communication overhead, and the extent to which learned representations improve the actual collection decisions. These are potential supporting capabilities rather than evidence that every such technique has been validated in MMS deployments.

#### 5.6.4. Comparative Analysis of Intelligent Coordination Methods

Learning-based MMS approaches differ in their decision variables, observation requirements, agent representations, and coordination mechanisms.

QAZP-based methods [68,69] primarily adapt network partitioning, whereas RLMP [24] focuses on sink movement decisions. These approaches improve the adaptability of specific collection decisions but do not necessarily learn explicit cooperation among multiple sinks. MARL-based approaches introduce interdependent decision-making among learning agents. Liu et al. [32] model physical mobile sinks as cooperative agents, whereas Rashid [70] coordinates clustering and routing functions. The latter demonstrates functional-agent cooperation rather than direct inter-sink coordination. Multi-UAV approaches [71,72] further demonstrate decentralized decision-making and distributed policy learning. However, federated parameter exchange alone does not guarantee coordinated movement or task allocation, and aerial mobility constraints may limit direct transferability to ground-based MMSs. Compared with conventional optimization, learning-based methods can reduce repeated online optimization through trained policies, but require training experience and computational resources. MARL additionally introduces agent interactions, potential non-stationarity, and coordination overhead. Neither improved training performance nor parameter sharing alone guarantees better data-collection performance.

The suitability of intelligent coordination methods therefore depends on network dynamics, observation availability, the number of physical sinks, training and inference costs, and communication requirements. Evaluations should consider collection latency, energy consumption, workload balance, scalability, and robustness alongside computational and coordination overhead.

## 6. Open Research Challenges and Future Research Directions

The five research dimensions reviewed in Section 5 reveal persistent gaps between algorithmic improvements and deployable MMS coordination. Practical systems must accommodate changing demand, coupled decisions, limited information exchange, heterogeneous collectors, and security threats. This section identifies these challenges and derives concrete evaluation requirements without introducing new dimensions into the taxonomy.

### 6.1. Practical Deployment Scenarios and Constraints

The feasibility of MMS-assisted data collection depends on application-specific mobility, connectivity, and service constraints that cannot be captured by energy consumption and average collection latency alone. The following examples illustrate deployment requirements rather than additional taxonomy categories. Traffic monitoring is constrained by road topology and the availability of communication infrastructure. Zhang et al. [76] investigated relay deployment in traffic WSNs, emphasizing connectivity and infrastructure placement. Although their study does not directly evaluate MMS coordination, it indicates that road accessibility and relay availability can affect collection-region assignment, sink trajectories, and delivery reliability.

Aerial collection introduces different restrictions, including obstacle avoidance, flight endurance, and communication security. He et al. [77] studied trajectory planning and resource allocation for secure communication in low-altitude networks with mixed obstacles. This work is not a direct MMS data-collection study, but its treatment of mobility and communication constraints is relevant to UAV-based collectors. Environmental monitoring and time-sensitive sensing introduce further requirements, such as uneven data generation, intermittent connectivity, and collection deadlines. These settings call for evaluations that explicitly model accessible travel paths, contact duration, platform energy budgets, communication reliability, and deadline compliance. Application relevance should be demonstrated through such deployment assumptions rather than inferred solely from improvements under idealized simulations.

### 6.2. Scalability and Dynamic Task Reassignment

Parallel collection can reduce the workload of individual sinks, but increasing the number of sinks also enlarges the set of interacting assignments, routes, and schedules. Static partitions may become inefficient when traffic demand, sensor energy, or sink availability changes. Fully centralized replanning can require costly state collection and computation, whereas independent local decisions can create duplicated service or uncovered regions.

A key research direction is event-triggered reassignment that responds to meaningful changes without continuously rebuilding the entire collection plan. Such mechanisms should specify reassignment triggers, temporary task ownership, and the treatment of buffered measurements during handover. Their scalability must be evaluated against the number of sensors and sinks, the frequency of environmental changes, reassignment completion time, and the additional control traffic.

### 6.3. Decision Coupling and Communication-Efficient Coordination

Task assignment, trajectory planning, routing, and scheduling influence one another. Reassigning sensors can change sink travel distances and contact opportunities, while a revised trajectory can invalidate established forwarding paths. Sequential optimization simplifies individual decisions but may preserve inefficient earlier choices. Explicitly coupled formulations can account for these dependencies, although discrete assignments, mobility restrictions, and resource constraints increase computational difficulty.

Future approaches should determine when joint or alternating updates justify their additional computational cost and when hierarchical decomposition is sufficient. Centralized and distributed control are separate implementation choices: both can support coupled decisions, but they differ in their information requirements and coordination overhead. Methods using local state, selective information sharing, or event-triggered synchronization should therefore be assessed under delayed and inconsistent observations. Evaluation must report optimization time, coordination messages, feasibility under deadlines, and sensitivity to outdated state information, rather than energy or latency improvements alone.

### 6.4. Learning Beyond Reinforcement Learning

RL and MARL support sequential adaptation and interdependent decision-making, but not every MMS problem requires a reinforcement-learning formulation. Supervised models may predict traffic demand, contact quality, or energy depletion; unsupervised learning may identify changing spatial demand patterns; and graph-based models may represent topology-dependent interactions. These capabilities can support assignment and planning without themselves constituting physical multi-sink coordination.

A research challenge is to determine which decisions benefit from prediction, representation learning, explicit optimization, or learned control, and how these components should interact. Federated model exchange can reduce centralized access to raw observations, but it does not alone ensure coordinated sink actions. Learning-based systems must therefore distinguish model-training architecture from the actual coupling of physical collectors’ decisions. Comparisons should include training data requirements, inference latency, communication cost, robustness to distribution shifts, and downstream collection performance.

### 6.5. Capability-Aware Heterogeneous Collectors

Many coordination models treat mobile sinks as interchangeable, although ground robots, road vehicles, and UAVs differ in reachable locations, travel time, endurance, communication range, and charging opportunities. Homogeneous assignments may consequently be infeasible or inefficient in mixed-platform deployments.

Capability-aware coordination should associate each collector with platform-specific movement, communication, and resource constraints. Feasible assignments may then combine ground-based collection in accessible areas with temporary aerial service in remote or disconnected regions. Heterogeneous-agent learning and hierarchical control offer possible solution mechanisms, but their value must be demonstrated through explicit task feasibility, contact scheduling, handover reliability, and mobility cost. Failure recovery is particularly important when a collector becomes unavailable before completing its assigned tasks.

### 6.6. Security-Aware Multi-Sink Coordination

MMS coordination depends on network-state reports, routing information, and inter-sink messages. Compromised nodes, falsified observations, or disrupted communication can therefore lead to incorrect assignments, inefficient routes, or interrupted data collection. Security mechanisms must protect both data forwarding and the coordination process.

Juwaied [78] proposed SecureFresh-WSN, combining anomaly detection, trust-aware clustering, and confidence-weighted routing. Although this work does not directly address cooperation among multiple physical mobile sinks, it provides relevant mechanisms for examining trust-sensitive MMS decisions. Future approaches should investigate authenticated state exchange, trust-aware task assignment, and attack-resilient reassignment under compromised sensors or unavailable collectors. Their evaluation should report detection quality and false alarms alongside delivery success, recovery delay, Age of Information (AoI), and security-induced coordination overhead.

### 6.7. Concrete Research Directions and Evaluation Criteria

Table 11 connects the preceding challenges to testable mechanisms and measurements. Its purpose is to identify comparable evaluation questions rather than rank methods developed for different system models.

Future evaluations should specify the number and capabilities of physical sinks, traffic dynamics, information availability, mobility and contact constraints, and the delivery objective. Experiments should also test disruptions such as sink failure, stale state information, and sudden demand changes. Reporting tail collection delay or AoI, successful delivery, task handover time, and control traffic per delivered measurement alongside network lifetime and mean latency would make the operational costs of coordination more transparent.

## 7. Conclusions

This survey has provided a comprehensive review of MMS-assisted data collection in WSNs, with particular emphasis on the coordination and optimization problems arising from the operation of multiple mobile collectors. Unlike conventional mobile-sink surveys that primarily focus on mobility patterns, routing strategies, or network lifetime, this work examines MMS research through a coordination-centered perspective. Five research dimensions were identified: task assignment and network partitioning, coordinated trajectory planning, multi-sink routing and data collection, cross-layer coupling and joint optimization, and intelligent multi-sink coordination. These dimensions were further organized into three complementary analytical levels: decision problems, decision coupling, and solution paradigms. This framework clarifies the relationships among individual MMS decisions and provides a basis for distinguishing sequentially coupled approaches from explicit joint optimization.

The reviewed literature shows that effective MMS operation requires more than independent sink movement or parallel data collection. Task assignment, trajectory planning, routing, and network organization are interdependent decisions whose interactions affect energy consumption, collection latency, mobility cost, and coordination overhead. Although existing studies have addressed several of these challenges through optimization and learning-based methods, important limitations remain in scalability, distributed coordination, and adaptation to dynamic network conditions. Learning-based approaches also require further investigation beyond RL and MARL, particularly in integrating prediction, graph-based learning, and distributed model adaptation with operational multi-sink coordination. Heterogeneous collectors and security-aware coordination introduce additional requirements involving capability-aware task allocation, reliable information exchange, and recovery from communication or node failures. Realistic deployment validation is also necessary to assess the effectiveness of these approaches under practical mobility and communication constraints.

Future MMS research should therefore focus on integrating decision-making across assignment, mobility, routing, and network control while maintaining scalability and operational reliability. Particular attention should be given to communication-efficient distributed coordination, adaptive task reassignment, heterogeneous mobile collectors, and security-aware data collection. Evaluation frameworks should also account for computational cost, coordination overhead, collection reliability, and application-specific requirements rather than relying solely on energy consumption or latency. Addressing these challenges will support the transition from parallel mobile data collection toward coordinated, adaptive, and intelligent MMS systems capable of operating in dynamic and heterogeneous sensing environments. 

## Figures and Tables

**Table 1:** Comparison of static-sink, single-mobile-sink, and multiple-mobile-sink architectures in WSNs.

Aspect	Static Sink	Single Mobile Sink	Multiple Mobile Sinks
Sink node	Fixed-location	Sequential mobile	Parallel and spatially distributed
Energy-hole mitigation	Limited	Improved through redistribution of forwarding load	Further improved through distributed collection and reduced forwarding distance
Scalability	Limited in large-scale networks	Moderate; constrained by tour length and collection capacity	High potential through parallel collection, but coordination complexity increases
Collection latency	Multi-hop distance and congestion	Sink tour length and revisit interval	Potentially reduced through parallel collection, but dependent on sink coordination
Task assignment	Generally not a primary issue	Usually unnecessary because one sink serves the entire network	Critical for determining which sink serves each node, cluster, or region
Network partitioning	Generally unnecessary	Optional for mobility or routing management	Important for defining service regions and distributing collection tasks
Trajectory planning	Not required	Single-sink path or trajectory optimization	Coordinated multi-sink trajectory planning is required
Routing	Routing toward fixed sink locations	Mobility-aware routing toward a moving sink	Multi-sink routing with dynamic sink selection and association
Inter-sink cooperation	Not applicable	Not applicable	Important for task redistribution, synchronization, information exchange, and cooperative collection
Optimization scope	Routing, clustering, and energy optimization	Joint routing–mobility or clustering–trajectory optimization	Joint assignment–routing–trajectory–scheduling optimization
Intelligent control	Typically focused on routing or network management	Adaptive mobility and trajectory control	Distributed and cooperative control using RL, MARL, and other intelligent approaches
Coordination overhead	Low	Low to moderate	Potentially high because of information exchange, synchronization, and cooperative decision-making
Main challenge	Energy imbalance and hotspot formation	Long collection tours, revisit delay, and limited collection capacity	Scalable coordination, joint optimization, and distributed decision-making

**Table 2:** Comparison of mobility models for MMS-assisted WSNs, including their effects on routing and task assignment.

Mobility Model	Movement Pattern	Advantages	Limitations	Routing Requirements	Task Assignment Strategies	Typical Applications
Random Mobility	Random or stochastic movement without a predefined trajectory	Simple implementation; low trajectory-planning overhead	Potentially high collection delay; poor energy balancing and coverage guarantees	Opportunistic forwarding or frequent sink-location updates	Assignments depending on proximity or expected contact opportunities	Highly dynamic environments
Predetermined Mobility	Fixed or periodically repeated trajectory	Predictable sink locations and contact times; simple routing and scheduling	Limited adaptation to changing network conditions	Relatively stable forwarding and reduced route-maintenance overhead	Fixed service regions based task allocation	Regular monitoring networks
Controlled and Optimized Mobility	Movement optimized according to network objectives	Improved energy balancing and network lifetime	Higher computational and signaling overhead	Routing decisions depending on optimized sink locations	Optimized collection points	Energy-constrained WSNs
Coordinated and Adaptive Mobility	Dynamic trajectories according to residual energy, traffic load, and network states	Adapts to time-varying network conditions	Frequent state updates and online decision-making overhead	Mobility-aware routing based on sink movement	Balanced dynamic task reassignment	Dynamic and heterogeneous WSNs
Hybrid Mobility	Combination of predetermined and adaptive sink trajectories	Balanced predictable collection and dynamic demand	Dependency on synchronization, handover, and overlap resolution	Scheduled forwarding with adaptive sink discovery	Stable baseline assignments	Mixed periodic and event-driven sensing

**Table 3:** Performance objectives and evaluation conditions of representative MMS studies.

Study	Performance Objectives	Network and Mobility Assumptions	Factors Affecting Performance
[16]	Collection latency, delivery success, and energy consumption	Multiple mobile sinks with alternative coordination strategies	Results depend on sink cooperation and available coordination information
[28]	Network lifetime	Coordinated sink mobility with discrete candidate configurations	Lifetime comparisons depend on mobility constraints and lifetime definition
[29]	Coverage and energy-related performance	Distributed consensus-based sink relocation	Coordination signaling and relocation conditions affect operational costs
[30]	Network lifetime and residual energy	Clustering-based regional assignment and planned collection trajectories	Performance depends on cluster geometry, trajectory construction, and network density
[31]	Forwarding redundancy and energy-related performance	Shared and bifurcated forwarding paths	Routing gains are not directly equivalent to mobility-cost reductions
[32]	Collection delay and collision-related performance	Learning-based cooperative path planning	Training conditions and agent model must be aligned before comparison

**Table 4:** Illustrative application-dependent priorities of MMS performance metrics.

Application	Reliability	Energy	Latency	Mobility Cost
Periodic environmental monitoring	H	H	M	M
Industrial/event monitoring	H	M	H	M
Large-area monitoring	H	H	M	H

H: high priority; M: moderate priority.

**Table 5:** Comparison of representative surveys from an MMS-oriented perspective.

Survey	Year	MMS	Partition	Mobility	Routing	Opt.	Coord.	Perspective
[33]	2014	Δ	–	Δ	Δ	–	–	Mobility-oriented data collection
[34]	2014	Δ	–	–	Δ	–	–	Distributed mobile-sink routing
[35]	2020	🗸	–	🗸	Δ	Δ	–	Mobility-based network lifetime
[36]	2021	🗸	Δ	Δ	Δ	–	–	Mobility patterns & communication
[37]	2022	🗸	–	Δ	Δ	–	–	General mobility in WSNs
[38]	2024	–	–	Δ	Δ	–	–	IoT data-collection architectures and protocols
[39]	2025	–	–	🗸	Δ	–	–	Mobile-sink collection and sensor recharging
This survey	2026	🗸	🗸	🗸	🗸	🗸	🗸	Coordination-centered, cross-layer, and intelligent perspective

**Table 6:** Comparison of Research Focuses and Analytical Limitations of Existing Surveys.

Survey	Primary Research Focus	MMS Analytical Coverage and Limitations
[33]	Sink mobility and data-collection mechanisms	Limited analysis of inter-sink task assignment and coordinated trajectory planning
[34]	Distributed routing and sink-location management	Limited analysis of task allocation and mobility-aware coordination across multiple sinks
[35]	Mobility-based network lifetime improvement	Limited emphasis on coordination overhead and multiple performance objectives
[36]	Mobility patterns and communication mechanisms	Limited analysis of dependencies among assignment, trajectory planning, and routing
[37]	General mobility mechanisms in WSNs	Limited analysis of MMS-specific cooperative optimization and distributed decision-making
[38]	IoT data-collection architectures and communication protocols	Limited analysis of MMS-specific assignment, coordinated mobility, and joint optimization
[39]	Mobile-sink data collection and sensor recharging	Limited emphasis on explicit coordination among multiple physical mobile sinks
This survey	Coordination-centered MMS analysis	Decision dependencies, cross-layer coupling, joint optimization, and intelligent multi-sink coordination

**Table 7:** Classification of representative MMS Studies by research objectives, coordination mechanisms, solution methods, and research dimensions.

Study	Research Objective	Coordination Mechanism	Solution Method	Dimensions
[16]	Coordinate data collection among multiple sinks	Coverage balancing and distributed sink coordination	Clustering and coordination heuristics	D1 (P), D2 (S)
[30]	Improve trajectory planning and network lifetime	Region-based allocation and rendezvous-point selection	K-means clustering and geometric path construction	D2 (P), D1 (S)
[28]	Maximize network lifetime through coordinated mobility	Joint sink configuration and movement scheduling	Linear programming and centralized/distributed heuristics	D2 (P), D4 (S)
[29]	Adapt service regions and sink relocation	Distributed consensus among mobile sinks	Consensus-based relocation and routing control	D1 (P), D3 (S)
[31]	Reduce redundant multi-sink forwarding	Shared forwarding paths and sink grouping	Distributed path merging and opportunistic forwarding	D3 (P), D1 (S)
[32]	Learn cooperative mobile-sink trajectories	Multi-agent cooperative decision-making	MADDPG with prioritized experience replay	D5 (P), D2 (S)

**Table 8:** Comparison of configuration-aware and runtime-adaptive assignment approaches for multiple mobile sinks.

Study	Partition	Mobility	Coordination	Main Objectives	Key Characteristics
[29]	Service regions associated with individual mobile sinks	Sink relocation triggered by coverage conditions	Distributed consensus among neighboring mobile sinks	Coverage maintenance, energy efficiency, routing efficiency	Adaptive relocation prevents local movement decisions
[41]	Number of clusters	Collection schedules and trajectories constructed for local sinks	Hierarchical local-sink/global-sink operation	Energy efficiency, scalability, collection efficiency	Joint clustering and the number of local mobile sinks
[42]	Fuzzy C-Means-based rendezvous-point grouping	ACO-based route construction	Linked assignment and trajectory planning	Short transmission distance, balanced path length, round-trip time	Integration of rendezvous-point and path planning
[43]	Energy-efficient clustering	Energy-aware routing	Artificial Bee Colony-based optimization	Energy consumption, cluster stability, coverage	Optimization-oriented multi-sink clustering
[44]	DBSCAN-based density-aware partitioning	ACO-based mobile-sink path planning	Density-aware assignment	Sink path length, mobility cost	Density-aware partitioning based on spatial node distribution; runtime reassignment not established
[45]	Virtual-unit-based partitioning	Regional movement with stopping-position selection	Mobility-aware hierarchical clustering and routing	Coverage, energy efficiency, routing stability	Extends region-based assignment to scenarios

**Table 9:** Comparison of representative multi-sink routing and data-collection approaches.

Study	Approach	Key Mechanism	Coordination Strategy	System Model	Design Limitations
[51]	Routing-oriented	K-medoids clustering and hybrid HHO–ACO sink mobility	Clustering–mobility coordination	Heterogeneous WSN	Primarily addresses hotspot mitigation rather than explicit joint routing control
[52]	Routing-oriented	K-medoids, HHO-based sink placement, and GWO-based routing	Coupled sink placement and routing	Heterogeneous WSN	Requires coordination among multiple optimization components
[53]	Routing-oriented	Reliability-aware ACO routing with alternative paths	Fault-tolerant route maintenance	WSN with critical forwarding points	Additional route maintenance is required when forwarding failures occur
[54]	Routing-oriented	Cluster-based hierarchical routing and mobile-sink collection	CH aggregation and sink-assisted delivery	Multi-mobile-sink WSN	Competing energy, latency, reliability, and throughput objectives
[55]	Routing-oriented	Combined Path Data Routing with shared forwarding segments	Shared-path construction and branching	Multiple mobile sinks	Benefits depend on opportunities for multi-destination path sharing
[31]	Routing-oriented	DCC, DVL, and opportunistic merged-path forwarding	Distributed multi-sink path sharing	Multiple mobile sinks	Effectiveness depends on forwarding and branching structures
[56]	Collection-oriented	Energy-aware rendezvous nodes and delay-constrained collection	Cluster- or geometry-based collection	Multiple mobile elements	Lifetime improvements must satisfy collection delay constraints
[57]	Collection-oriented	Mobile-sink collection scheduling and Cloud uploading	Multi-sink collection scheduling	WSN-to-Cloud collection	Energy savings depend on uploading latency requirements
[58]	Collection-oriented	Voronoi collection zones and DCF scheduling	Mobile fog-element coordination	Fog-assisted WSN-to-Cloud	Performance depends on scheduling and communication delays
[59]	Collection-oriented	DADCA and distributed information exchange	Encounter-based task redistribution	Multiple UAV collectors	Coordination depends on UAV encounters and communication opportunities

**Table 10:** Methodological characteristics and operating conditions of cross-layer MMS approaches.

Methodological Aspect	Information Requirements	Computational/ Communication Costs	Suitable Conditions	Main Limitations
Centralized decision-making	Global or aggregated network state	Central optimization and state collection	Networks with reliable control infrastructure	Decision latency, stale information, controller dependence
Distributed decision-making	Local observations and information exchange	Local computation and recurring coordination messages	Large-scale or infrastructure-limited networks	Inconsistent state, synchronization overhead
Heuristic/ metaheuristic search	Defined objectives, constraints, and algorithm parameters	Iteration-dependent computation; communication varies by implementation	Complex problems where exact solutions are impractical	Parameter sensitivity and limited optimality guarantees
Adaptive operation	Repeated observation of traffic and network conditions	Monitoring, recomputation, and reassignment signaling	Time-varying demand and heterogeneous network conditions	Control overhead and decision instability

**Table 11:** Research challenges, candidate directions, and evaluation criteria for future MMS coordination.

Challenge	Limitation	Research Direction	Evaluation Criteria
Deployment constraints	Idealized movement and connectivity assumptions	Road-flight-feasible trajectories and contact-aware collection	Deadline satisfaction, contact success, mobility energy, reliability
Dynamic assignment	Fixed service regions and delayed recovery	Event-triggered reassignment with explicit task handover	Reassignment delay, missed collections, control messages
Coupled decisions	Sequential stages may preserve inefficient upstream choices	Coupled or alternating optimization with explicit constraints	Feasibility, lifetime, tail latency, computation time
Distributed coordination	Stale local state and excessive information exchange	Selective updates and communication-budgeted coordination	Messages per delivered measurement, scalability, reliability
Intelligent coordination	Training burden, partial observations, uncertain transferability	Prediction-assisted planning and communication-aware MARL	Training/inference cost, robustness, collection performance
Heterogeneous collectors	Different accessibility, endurance, and contact constraints	Capability-aware allocation and cross-platform recovery	Task completion, AoI, recovery time, mobility cost
Security-aware coordination	Manipulated observations and untrusted coordination messages	Trust-aware assignment and attack-resilient information exchange	Detection/false-alarm rates, delivery, AoI, recovery overhead

## Data Availability

Data is contained within the article.
